# FUT2 enhances anti-tumor immunity in pancreatic cancer radiotherapy by driving FBXO2-mediated degradation of NR2F2

**DOI:** 10.1038/s41419-025-08378-2

**Published:** 2025-12-23

**Authors:** Junguo Chen, Yun Chen, Zhuobin Lin, Zhihuang Liang, Hua Yu, Cheng Wang, Hui Peng, Xiongjun Wang, Kunhua Hu

**Affiliations:** 1 Precise Genome Engineering Centre, School of Life Sciences, Guangzhou University, Guangzhou, China; 2 Guangdong Provincial Key Laboratory of Liver Disease Research, The Third Affiliated Hospital of Sun Yat-sen University, Guangzhou, Guangdong China

**Keywords:** Cancer microenvironment, Cell death

## Abstract

Pancreatic ductal adenocarcinoma (PDAC) radiotherapy (RT) resistance is frequently mediated by an immunosuppressive tumor microenvironment (TIME). Utilizing an in vivo CRISPR-Cas9 metabolic enzyme screen, we identified fucosyltransferase 2 (FUT2) as a potent non-catalytic enhancer of RT response. Mechanistically, FUT2 scaffolds the E3 ubiquitin ligase FBXO2, facilitating K362 site-specific ubiquitination and proteasomal degradation of the transcription factor NR2F2. This degradation suppresses expression of the immunosuppressive factor Lipocalin-2 (LCN2), which drives CD8⁺ T cell exhaustion and impedes NK cell infiltration, fostering a radioresistant TIME. Interestingly, we observed that RT could reduce FUT2 transcript levels via an METTL14-mediated m⁶A RNA methylation, while NR2F2 was identified to transcriptionally upregulate METTL14, establishing a feedforward inhibitory loop that sustains FUT2 suppression. Clinically, FUT2 expression positively correlates with CD8⁺ T cell infiltration and prolonged survival in RT-treated PDAC patients. Preclinically, combining RT with LCN2-neutralizing antibodies elicited synergistic anti-tumor immunity. These results unveil FUT2 as a regulator of PDAC radiosensitivity via the FUT2-FBXO2-NR2F2-LCN2 axis, offering a promising therapeutic target to overcome RT resistance.

## Introduction

Pancreatic ductal adenocarcinoma (PDAC) is one of the most lethal malignancies, with a 5-year survival rate of less than 10% [1]. Current clinical management typically involves surgical resection followed by chemotherapy and radiotherapy (RT). While RT has shown therapeutic efficacy in various cancers, its effectiveness as an adjuvant treatment in PDAC remains inconsistent [2–4]. This is primarily due to the establishment of an immunosuppressive tumor immune microenvironment (TIME) in PDAC, which confers resistance to conventional therapies, including radiotherapy [5]. Thus, improving radiosensitivity in PDAC remains a critical challenge for treating this highly refractory malignancy.

RT has been shown to enhance anti-tumor immunity through mechanisms such as upregulating MHC class I molecules on tumor cells, increasing T-cell recognition of tumor antigens [6], and triggering immunogenic cell death, which triggers T cell-mediated anti-tumor responses [7]. However, RT can also paradoxically contribute to the formation of an immunosuppressive TIME, allowing tumors to evade immune surveillance. Increased PD-L1 expression [8], enhanced recruitment and activation of regulatory T cells [9], and lymphocyte depletion or exhaustion [10, 11] are common consequences of RT treatment. To fully harness the immune-enhancing potential of RT, it is crucial to elucidate the molecular factors driving immune resistance.

Metabolic plasticity is increasingly recognized as a key feature of tumor progression and therapeutic resistance. Through metabolic reprogramming, cancer cells not only meet their own growth demands but also reshape the TIME, contributing to resistance against both radiotherapy and chemotherapy [12]. In this context, we aimed to identify novel metabolic targets using in vivo metabolic CRISPR-Cas9 knockout screening and elucidate the molecular mechanisms underlying RT-induced immune escape in PDAC. Specifically, we uncovered fucosyltransferase 2 (FUT2), a member of the fucosyltransferase family, catalyzes the transfer of fucose residues to terminal galactose moieties on cell-surface glycoproteins and glycolipids, thereby modulating cell-cell interactions [13]. Surprisingly, we found that FUT2 enhances RT-induced anti-tumor immunity independently of its enzymatic activity, instead relying on its protein abundance. Such enzyme-independent functions are increasingly recognized in metabolic enzymes like FBP1 and HK2, which participate in complex cellular processes beyond their classical metabolic roles [14, 15].

The Skp1-Cullin1-F-box (SCF) complex, a critical member of the RING-finger family of E3 ubiquitin ligases, comprises the scaffold protein Cullin1, the adaptor protein SKP1, RING-finger proteins Rbx1/2, and an F-box protein responsible for substrate recognition and ubiquitination [16]. Among these, FBXO2 mediates the ubiquitination and proteasomal degradation of multiple target proteins, playing roles in cancer and neurological diseases [17, 18]. Given their dual roles in both promoting and suppressing tumorigenesis [19], F-box proteins present potential therapeutic targets depending on the tumor context.

In this study, we identified FUT2 as a metabolic enzyme significantly downregulated following RT in PDAC cells. Constitutive overexpression of FUT2 increased the infiltration of cytotoxic CD8^+^ T cells and NK cells into the TIME, mitigating RT-induced immune escape. These findings reveal a novel mechanism by which PDAC tumors remodel their immunosuppressive TIME to mediate radioresistance, offering potential therapeutic targets for improving the efficacy of radiotherapy.

## Results

### FUT2 synergistically enhances pancreatic cancer cell death following radiotherapy

**Fig. 1:**
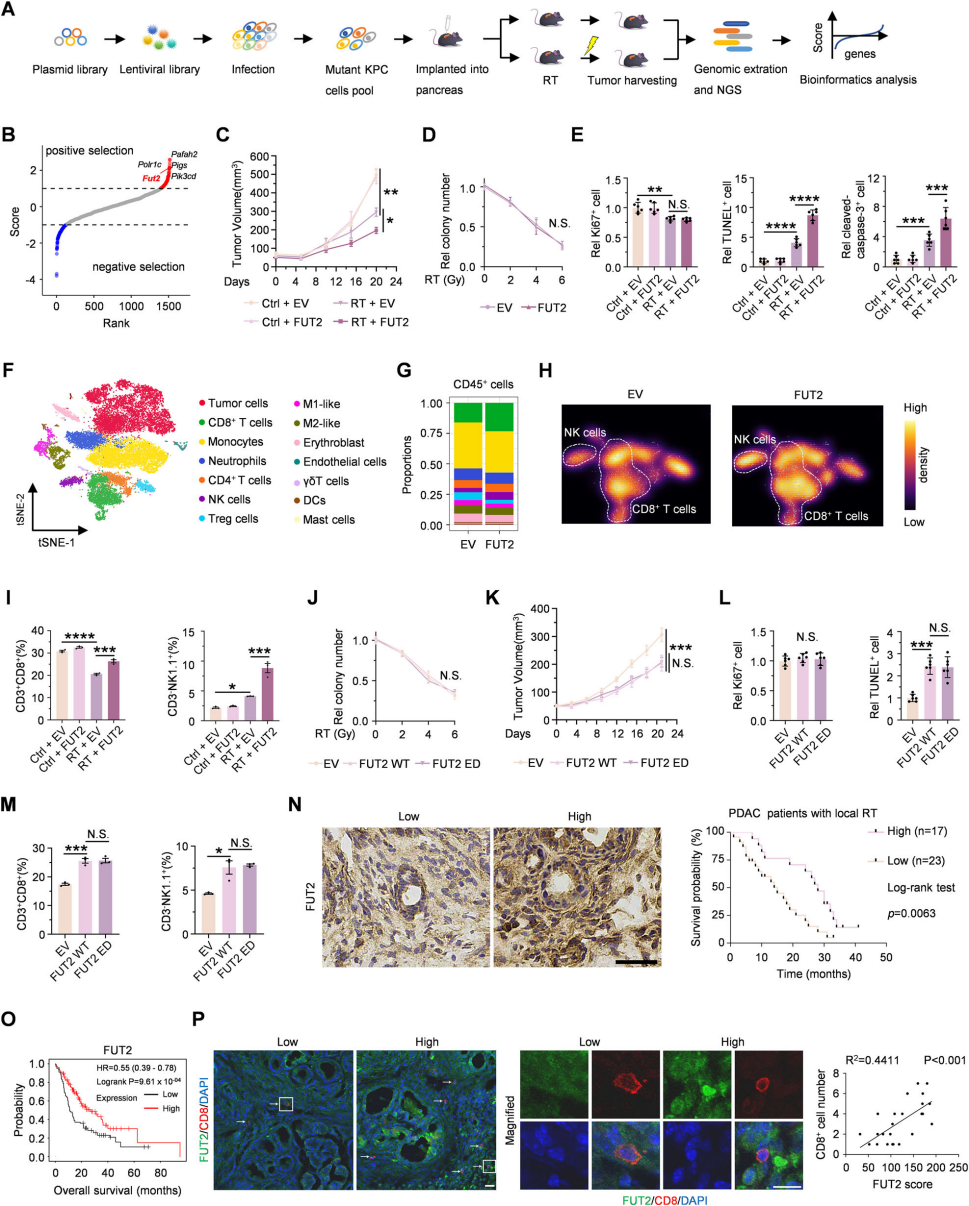
FUT2 synergistically enhances pancreatic cancer cell death following radiotherapy. **A** Schematic illustration of the workflow for in vivo CRISPR knockout screening. **B** The enrichment of positive and negative candidates in the CRISPR screen. **C** KPC cells overexpressing EV or FUT2 were subcutaneously injected to C57BL/6 mice that were then treated with or without RT at 14th day (*n* = 6). Tumor volumes were measured. EV, empty vector. RT, radiotherapy. **D** Quantification of colony formation in KPC cells overexpressing EV or FUT2 following exposure to varying doses of RT. **E** KPC cells overexpressing EV or FUT2 were subcutaneously injected to C57BL/6 mice that were then treated with or without RT at 14th day. Quantifications of tumor proliferative and dead index determined by Ki67, TUNEL, and cleaved caspase-3 staining are shown. **F**–**H** KPC cells overexpressing EV or FUT2 were subcutaneously injected to C57BL/6 mice that were then treated with RT at 14th day. The tumors were subjected to single-cell sequencing and the subtypes of immune cells are shown. **I** KPC cells overexpressing EV or FUT2 were subcutaneously injected to C57BL/6 mice that were then treated with or without RT at 14th day. Proportions of the cytotoxic T lymphocytes (CD3^+^CD8^+^) and NK cells (CD3^-^NK1.1^+^) in tumors were analyzed by flow cytometry. **J** Quantification of colony formation in KPC cells overexpressing EV, FUT2 WT or ED following exposure to varying doses of RT. KPC cells overexpressing EV, FUT2 WT or ED were subcutaneously injected to C57BL/6 mice that were then treated with RT at 14th day. Tumor volume (**K**), quantifications of tumor proliferative and dead index determined by Ki67 and TUNEL staining (**L**), proportions of the cytotoxic T lymphocytes and NK cells (**M**) analyzed by flow cytometry are shown. **N** Forty patients with locally advanced PDAC scheduled for local RT were divided into two groups based on the optimal cutoff of FUT2 IHC score, classified as high or low expression. Survival curves were then generated to compare outcomes between the two groups. Scale bar, 50 μm. **O** Kaplan–Meier survival analyses of low and high FUT2 expression in pancreatic cancer patients via a Kaplan–Meier plotter database. **P** CD8^+^ T cell and FUT2 of the pancreatic cancer patients were measured using immunofluorescence co-staining and analyzed by calculating the Pearson correlation coefficient. Scale bar, 20 μm. ^*^p < 0.05, ^**^p < 0.01; ^***^p < 0.001; ^****^p < 0.0001; N.S., not significant; two-way ANOVA [(**C**–**E**) and (**I**–**M**)], log-rank test (**N**) or Pearson correlation coefficient test (**P**).

To identify novel metabolic targets that potentiate RT efficacy in pancreatic cancer, we conducted an in vivo CRISPR-Cas9 knockout screening using a murine metabolic enzyme library. KPC cells infected with this library were orthotopically implanted into the pancreas of C57BL/6 mice and subsequently irradiated (Fig. 1A). We found that FUT2 was ranked on the top of the single-guide RNAs (sgRNAs) targeting metabolic genes in the tumor tissues after RT, implicating FUT2 as a potential enhancer of RT response (Figs. 1B and S1A–S1C). Further experiments using subcutaneous KPC tumor models showed that FUT2 overexpression alone did not alter tumor growth but markedly sensitized tumors to RT-induced suppression (Figs. 1C and S1D–S1F). Critically, in vitro assays confirmed that FUT2 overexpression did not directly impact KPC cell viability following irradiation compared to control cells (Figs. 1D and S1G). Immunohistochemical (IHC) analysis using Ki67 and TUNEL staining revealed that FUT2 overexpression did not significantly affect cell proliferation but notably increased tumor cell death following RT. Additionally, IHC for cleaved caspase-3 confirmed increased apoptosis in FUT2-overexpressing tumors, and FUT2 IHC verified that overexpression was maintained across the experiment (Figs. 1E and S1H). Given these observations, we hypothesized that FUT2 potentiates the anti-tumor effects of RT through modulation of TIME. Single-cell RNA sequencing data indicated increased proportions of tumor-infiltrating CD8^+^ T cells and NK cells in FUT2-overexpressing tumors (Fig. 1F–H). These findings were further corroborated by flow cytometric analysis of immune cell populations extracted from tumor tissues, demonstrating enhanced infiltration of CD8^+^ T cells and NK cells upon FUT2 overexpression (Figs. 1I and S1I).

FUT2 catalyzes the addition of L-fucose residues to terminal galactose moieties on glycoproteins and glycolipids [13], regulating processes such as cell-cell interactions and proliferation, potentially influencing TIME remodeling [20]. To determine whether the enzymatic activity of FUT2 is essential for its therapeutic synergy with RT, we generated a enzymatically dead mutant (FUT2 ED) and confirmed its loss of enzymatic activity in KPC cells (Fig. S1J). Following irradiation, colony formation assays in vitro revealed comparable cell viability among cells stably overexpressing FUT2 WT or ED, and vector control (Figs. 1J, and S1K, L). However, both FUT2 WT and ED significantly inhibited in vivo tumor growth following RT, with no notable difference between the two groups (Figs. 1K and S1L–S1N). Immunostaining assays further confirmed that FUT2 WT and FUT2 ED comparably increased RT-induced cell death without significantly affecting proliferation relative to control (Figs. 1L and S1O). Flow cytometric analysis showed that both FUT2 WT and ED enhanced CD8^+^ T and NK cells infiltration, similarly, indicating enzyme-independent activity (Figs. 1M and S1P). To further validate these findings, we established FUT2-knockdown KPC cells using short hairpin RNA (shFUT2) and observed significantly enhanced tumor growth following RT compared to non-targeting controls (shNT). Reintroduction of either shRNA-resistant wild-type FUT2 (rFUT2 WT) or rFUT2 ED completely abrogated this phenotype, with both groups showing comparable suppression of tumor growth (Fig. S1Q–S1S). These results reinforce that FUT2-mediated radiosensitization occurs independently of its catalytic activity.

To assess clinical relevance, tumor samples from locally advanced PDAC patients receiving RT were categorized into high- and low-FUT2 expression groups based on immunohistochemical staining intensity. Kaplan–Meier survival analyses revealed that higher FUT2 expression correlated significantly with improved patient outcomes post-RT (Fig. 1N), consistently with the independent analysis of pancreatic cancer survival data from the Kaplan–Meier Plotter database (Fig. 1O). Importantly, FUT2 levels positively correlated with CD8^+^ T cell infiltration in PDAC tissues (Fig. 1P), supporting its role in alleviating immunosuppression within the TIME.

### FUT2 enhances radiotherapy efficacy by suppressing LCN2 expression

**Fig. 2:**
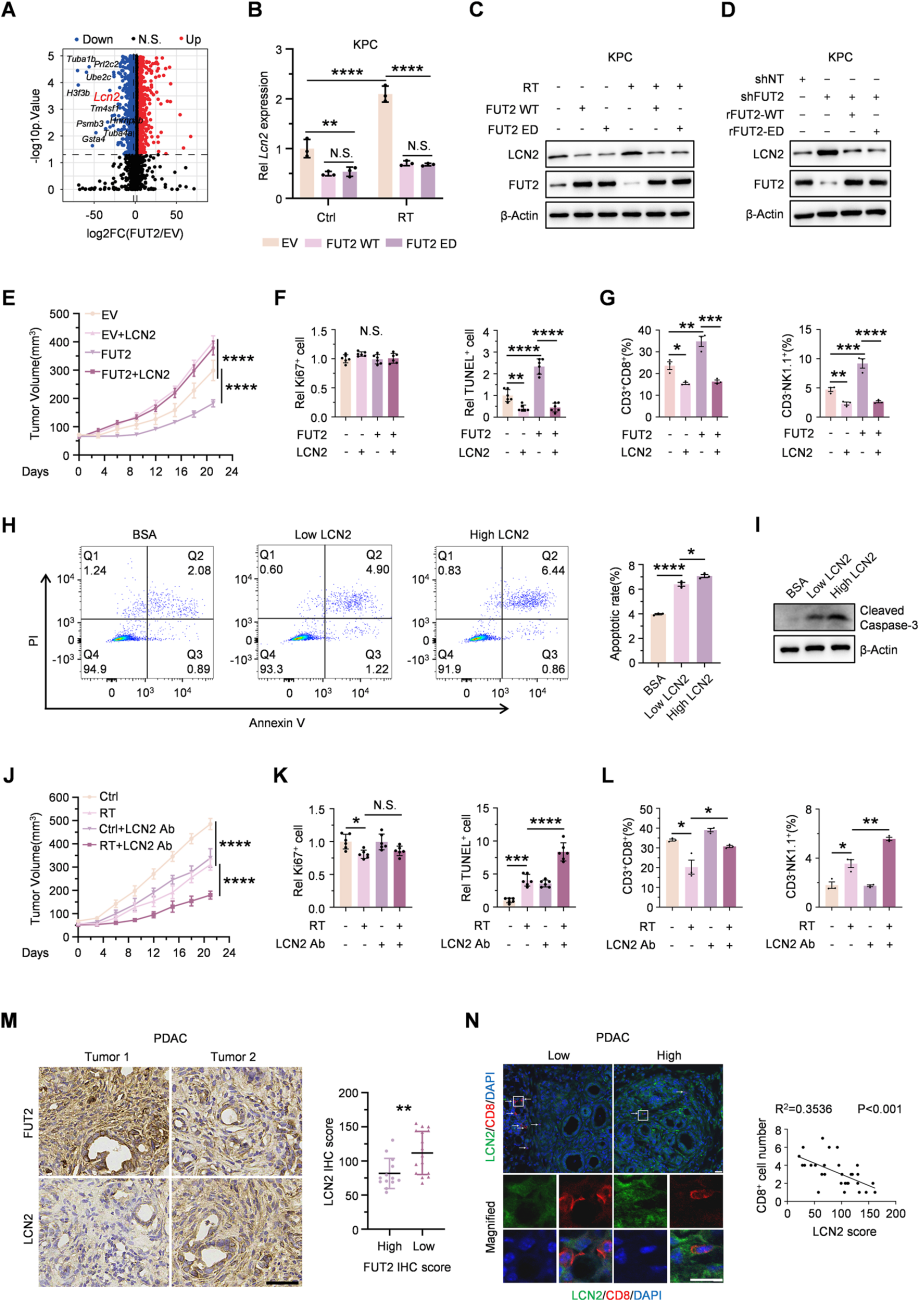
FUT2 enhances radiotherapy efficacy by suppressing LCN2 expression. **A** RNA profiling was performed on KPC cells overexpressing EV or FUT2 12 h post-RT. The differentially expressed genes (DEGs) were analyzed, and the top 10 DEGs are presented. Expression levels of *LCN2* mRNA (**B**) and protein (**C**) were measured in KPC cells overexpressing EV, FUT2 WT, or ED in the absence or presence of RT, with RNA and protein samples collected at 12 and 24 h post-RT, respectively. **D** Immunoblotting analyses of indicated proteins in KPC cells expressing shNT, shFUT2, and shFUT2 rescued with rFUT2 WT or ED. **E**–**G** Subcutaneous transplantation of EV- or FUT2-overexpressing KPC cells into C57BL/6 mice that were then treated with RT at 14th day. Exogenous LCN2 supplementation was performed as described in the supplementary methods (*n* = 8). Tumor volume (**E**), quantifications of tumor proliferative and dead index determined by Ki67 and TUNEL staining (**F**), proportions of the cytotoxic T lymphocytes and NK cells analyzed by flow cytometry (**G**) are shown. **H**, **I** Flow cytometry and immunoblotting were performed to test the apoptosis of CD8^+^ T cells induced by exogenous LCN2 supplementation. **J**–**L** KPC cells were subcutaneously transplanted into C57BL/6 mice that were then treated with RT or LCN2 neutralizing antibodies as described in the supplementary methods (*n* = 8). Tumor volume (**J**), quantifications of tumor proliferative and dead index determined by Ki67 and TUNEL staining (**K**), proportions of the cytotoxic T lymphocytes and NK cells analyzed by flow cytometry (**L**) are shown. **M** IHC staining for LCN2 was obtained and analyzed between two groups divided by IHC score of FUT2 in PDAC tissues. Scale bar, 50 μm. **N** CD8^+^ T cell and LCN2 were measured using immunofluorescence co-staining and analyzed by calculating the Pearson correlation coefficient. Scale bar, 20 μm. ^*^p < 0.05; ^**^p < 0.01; ^***^p < 0.001; ^****^p < 0.0001; N.S., not significant; two-way ANOVA [(**B**) and (**E**–**H**) and (**J**–**L**)] or Two tailed Student’s *t* test (**M**).

To investigate the mechanism by which FUT2 modulates the immunosuppressive TIME in pancreatic cancer, we conducted RNA sequencing on KPC cells overexpressing FUT2 versus empty vector (EV) following RT treatment. LCN2 emerged among the top ten significantly downregulated genes, as the sole secretory protein (Fig. 2A). Despite established roles in tumor iron metabolism and T cell differentiation or dysfunction, whether LCN2 contributes to RT-induced TIME remodeling remained undefined [21–23]. Quantitative RT-PCR analysis confirmed that RT markedly increased LCN2 transcription, whereas overexpression of either FUT2 WT or ED comparably effectively suppressed this induction (Figs. 2B and S2A). Notably, genetic knockdown of FUT2 further enhanced RT-induced LCN2 mRNA accumulation in both KPC and PANC-1 cells (Fig. S2B). Mirroring these transcriptional changes at the protein level, FUT2 overexpression significantly attenuated RT-induced LCN2 expression in both cell lines (Figs. 2C and S2C). Crucially, FUT2 depletion markedly elevated basal LCN2 levels, and this elevation was comparably reversed by reconstitution with either rFUT2 WT or ED (Figs. 2D and S2D), further supporting an enzyme-independent mechanism of LCN2 regulation.

To confirm the functional importance of FUT2-mediated inhibition of LCN2 in enhancing radiosensitivity, recombinant LCN2 protein was injected into subcutaneous tumors derived from KPC cells overexpressing FUT2. Exogenous LCN2 protein abrogated the radiosensitizing effect conferred by FUT2 overexpression (Figs. 2E, and S2E and S2F). Immunohistochemical staining for Ki67 and TUNEL indicated that LCN2 supplementation did not alter tumor proliferation but significantly enhanced tumor cell survival post-irradiation (Figs. 2F and S2G). Flow cytometry further confirmed that LCN2 supplementation reversed the increase in tumor-infiltrating CD8^+^ T cells and NK cells induced by FUT2 overexpression (Figs. 2G and S2H). Given previous evidence linking LCN2 to T cell differentiation or apoptosis [21, 22], we performed apoptosis assays on activated CD8^+^ T cells and found dose-dependent induction of apoptosis, corroborated by increased cleaved caspase-3 on immunoblots (Fig. 2H, I).

Next, we evaluated the therapeutic potential of neutralizing LCN2 by administering LCN2-neutralizing antibodies intravenously in mice bearing KPC-derived tumors. ELISA confirmed on-target activity of the treatment, with a significant reduction in secretory LCN2 levels (Fig. S2I). RT alone suppressed tumor growth, and combination treatment with LCN2-neutralizing antibodies significantly enhanced this suppression (Figs. 2J, and S2J, K). Immunohistochemical analysis confirmed that combined treatment promoted tumor cell apoptosis without affecting cell proliferation significantly (Figs. 2K and S2L). Flow cytometric analysis demonstrated increased infiltration of CD8^+^ T cells and NK cells, further confirming attenuation of the immunosuppressive TIME following combined therapy (Figs. 2L and S2M). Thus, targeting LCN2 in combination with RT effectively mitigates immunosuppression and enhances anti-tumor efficacy in pancreatic cancer.

Clinically, we performed immunohistochemical staining for LCN2 in pancreatic cancer patient samples stratified according to FUT2 expression intensity. Tumors with higher FUT2 expression exhibited reduced LCN2 protein levels (Fig. 2M). Additionally, LCN2 expression inversely correlated with CD8^+^ T cells infiltration (Fig. 2N), further supporting the role of LCN2 in mediating RT resistance.

### FUT2 represses LCN2 transcription by facilitating NR2F2 destabilization

**Fig. 3:**
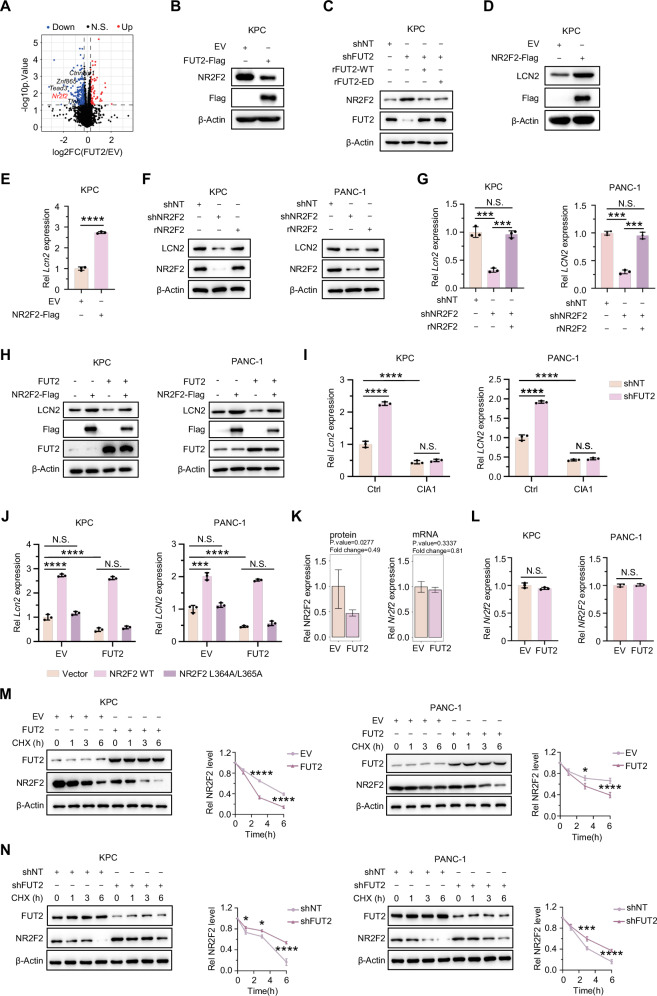
FUT2 represses LCN2 transcription by facilitating NR2F2 destabilization. **A** LC–MS analysis was performed on KPC cells overexpressing EV or FUT2 at 12 h post-RT, revealing five major transcription-related factors. **B**, **C** Immunoblotting analyses of indicated proteins in KPC cells with the indicated genetic manipulation. Expression levels of LCN2 protein (**D**) and mRNA (**E**) were measured in KPC cells overexpressing EV or NR2F2-Flag. Expression levels of LCN2 protein (**F**) and mRNA (**G**) were measured in KPC and PANC-1 cells expressing shNT. shNR2F2 and shNR2F2 rescued with rNR2F2. **H** Immunoblotting analyses of indicated proteins in KPC and PANC-1 cells overexpressing EV, EV with NR2F2-Flag, FUT2, or FUT2 with NR2F2-Flag. **I** Expression levels of LCN2 mRNA were measured in KPC and PANC-1 cells expressing shNT or shFUT2 and subsequently treated with or without CIA1 (4 μM) for 12 h. **J** Expression levels of LCN2 mRNA were measured in KPC and PANC-1 cells overexpressing either EV or FUT2 following transfection with Vector, NR2F2 WT, or NR2F2 L364A/L365A. **K** Comprehensive comparison between RNA-seq and LC-MS results of NR2F2. **L** Expression levels of NR2F2 mRNA were measured in KPC and PANC-1 cells overexpressing EV or FUT2. **M** Immunoblotting analyses of indicated proteins in KPC and PANC-1 cells overexpressing EV or FUT2 and subsequently treated with CHX for indicated time. **N** Immunoblotting analyses of indicated proteins in KPC and PANC-1 cells expressing shNT or shFUT2 and subsequently treated with CHX for indicated time. ^*^P < 0.05; ^***^P < 0.001; ^****^P < 0.0001; N.S., not significant; Two tailed Student’s *t* test [(**E**, **L**)]. or two-way ANOVA [(**G**), (**I**, **J**) and (**M**, **N**)].

To systematically identify regulators responsible for FUT2-dependent LCN2 suppression, we performed LC-MS/MS-based proteomic profiling of KPC cells overexpressing EV or FUT2 following RT treatment. Among the top differentially expressed transcriptional regulators screened, including NR2F2, TFEB, TEAD3, CTNNBIP1, and ZNF865 (Fig. 3A), subsequent immunoblot analysis in KPC and PANC-1 cells showed that only NR2F2 exhibited statistically significant downregulation upon FUT2 overexpression compared to control cells (Figs. 3B, and S3A, B). Consistent with this regulation, FUT2 knockdown substantially increased NR2F2 protein levels, which were comparably rescued by reconstitution with either rFUT2 WT or ED (Figs. 3C and S3C). Conversely, forced NR2F2 expression markedly elevated both protein and mRNA levels of LCN2 (Figs. 3D, E, and S3D, E). Complementary genetic evidence demonstrated that NR2F2 knockdown significantly reduced LCN2 expression at transcriptional and translational levels, an effect reversed by reintroduction of shRNA-resistant NR2F2 (rNR2F2) (Fig. 3F, G).

To further confirm the role of NR2F2 in FUT2-mediated suppression of LCN2, we reintroduced Flag-tagged NR2F2 into FUT2-overexpressing KPC and PANC-1 cells. This experiment demonstrated that NR2F2 overexpression effectively reversed FUT2’s suppressive effect on LCN2 expression (Fig. 3H). Additionally, FUT2 silencing significantly increased LCN2 transcription, but this effect was effectively reversed by CIA1, a validated NR2F2 inhibitor [24, 25], in multiple cancer cells (Fig. 3I). To verify NR2F2’s direct transcriptional regulation of LCN2, we transduced cells with either wild-type NR2F2 (NR2F2-WT) or a transcriptionally inactive mutant (NR2F2-L364A/L365A) [26]. FUT2 overexpression suppressed LCN2 transcription, whereas reintroducing NR2F2-WT restored LCN2 expression and counteracted FUT2-mediated suppression. In contrast, the NR2F2-L364A/L365A mutant failed to rescue LCN2 expression, underscoring NR2F2’s transcriptional activity as essential for LCN2 induction (Fig. 3J).

Given that FUT2 did not significantly alter NR2F2 mRNA levels, as confirmed by comparative analysis of RNA-seq and LC-MS/MS data as well as qRT-PCR analysis (Fig. 3K, L), we hypothesized a post-transcriptional regulatory mechanism. Indeed, assessment of NR2F2 protein stability showed that FUT2 overexpression markedly shortened the protein half-life of NR2F2 in both KPC and PANC-1 cells (Fig. 3M). Conversely, FUT2 knockdown significantly prolonged NR2F2 half-life (Fig. 3N). These results demonstrate that FUT2 reduces the transcriptional expression of LCN2 by unstabilizing NR2F2.

### FUT2 promotes NR2F2 degradation through ubiquitination at lysine 362 via the proteasomal pathway

**Fig. 4:**
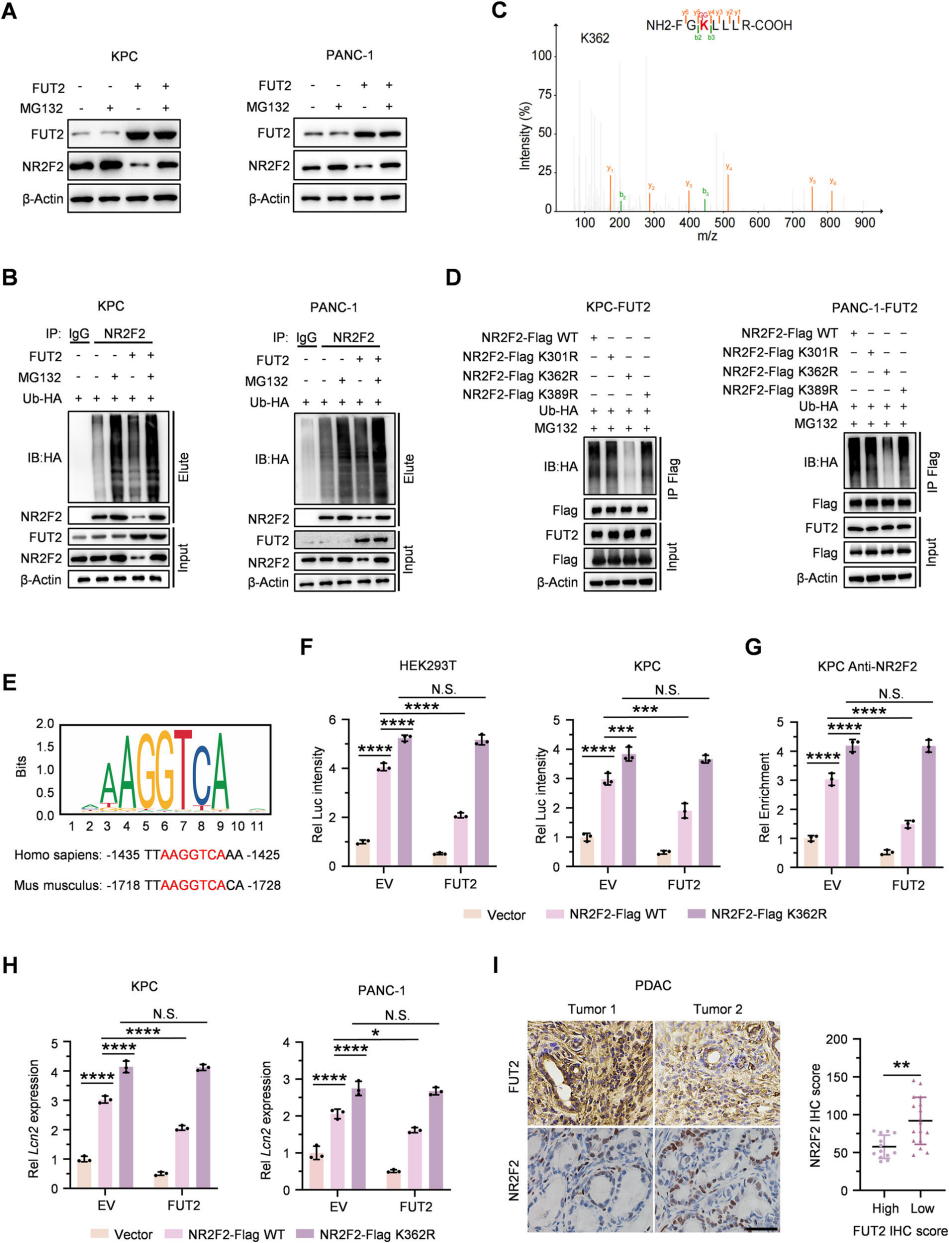
FUT2 promotes NR2F2 degradation through ubiquitination at lysine 362 via the proteasomal pathway. **A** Immunoblotting analyses of indicated proteins in KPC and PANC-1 cells overexpressing EV or FUT2 and subsequently treated with or without MG132 (10 μM) before harvesting. **B** KPC and PANC-1 cells overexpressing EV or FUT2 were transfected with Ub-HA and then treated with or without MG132 (10 μM) before harvesting. Immunoprecipitation and immunoblotting analyses were performed with indicated proteins. **C** NR2F2-Flag was subjected to LC‒MS and identified three potential ubiquitination sites. **D** KPC and PANC-1 cells overexpressing FUT2 were transfected with Ub-HA and NR2F2-Flag WT or mutants, subsequently treated with MG132 (10 μM) before harvesting. Immunoprecipitation and immunoblotting analyses were performed with indicated proteins. **E** Screening of the LCN2 promoter region identified one of the potential NR2F2 binding motif sites in both humans and mice. **F** Dual-luciferase assay was performed to measure LCN2 promoter activity in HEK293T and KPC cells co-transfected with FUT2 and either NR2F2-Flag WT or NR2F2-Flag K362R. **G** ChIP-qPCR validation of LCN2 in KPC cells overexpressing either EV or FUT2 following transfection with Vector, NR2F2-Flag WT or NR2F2-Flag K362R and immunoprecipitated with anti-NR2F2 antibody. IgG was used as a blank control. **H** Expression levels of LCN2 mRNA were measured in KPC and PANC-1 cells overexpressing either EV or FUT2 following transfection with Vector, NR2F2-Flag WT or NR2F2-Flag K362R. **I** IHC staining for NR2F2 was obtained and analyzed between two groups divided by IHC score of FUT2 in PDAC tissues. Scale bar, 50 μm. ^*^P < 0.05; ^**^P < 0.01; ^****^P < 0.0001; N.S., not significant; two-way ANOVA (**F**–**H**) or Two tailed Student’s *t* test (**I**).

Given previous reports indicating that NR2F2 undergoes proteasomal degradation mediated by ubiquitination [27], and we observed the increased NR2F2 protein half-life upon FUT2 depletion (Fig. 3N), thus, we want to examine whether FUT2 modulates NR2F2 stability through a similar mechanism. As a result, proteasome inhibitor MG132 significantly increased NR2F2 protein levels and partially reversed FUT2-induced reduction of NR2F2 (Fig. 4A), suggesting that FUT2 potentially regulates NR2F2 stability via the ubiquitin-proteasome pathway. FUT2 overexpression markedly enhanced NR2F2 ubiquitination and concurrently reduced NR2F2 protein abundance, effects that were intensified by MG132 treatment, leading to restoration of NR2F2 levels (Fig. 4B). Additionally, we observed that NR2F2’s instability specifically involved K48-linked ubiquitination (Fig. S4A).

To pinpoint NR2F2 ubiquitination sites, LC-MS/MS analysis of immunoprecipitated NR2F2 from FUT2-overexpressing cells identified three candidate ubiquitinated lysine residues: K301, K362, and K389 (Figs. 4C and S4B). Mutation of each site individually to arginine (NR2F2-K301R, NR2F2-K362R, NR2F2-K389R) in FUT2-overexpressing KPC and PANC-1 cells revealed that the K362R mutation significantly decreased ubiquitination compared to wild-type NR2F2 or other mutants, resulting in the stability of NR2F2 protein (Fig. 4D). Thus, FUT2-mediated ubiquitination and subsequent degradation of NR2F2 primarily occurs at lysine 362.

Next, considering the transcriptional regulatory axis involving NR2F2 and LCN2, bioinformatics analysis of the LCN2 promoter region via the JASPAR database revealed a high-confidence NR2F2-binding motif conserved in humans and mice (Fig. 4E). Luciferase reporter assays demonstrated that NR2F2 overexpression robustly activated LCN2 promoter activity, an effect markedly suppressed by FUT2 co-expression. Critically, the NR2F2-K362R mutation abolished FUT2’s inhibitory effect, maintaining high luciferase activity comparable to NR2F2-K362R expression alone, exceeding levels induced by NR2F2-WT (Fig. 4F). Chromatin immunoprecipitation (ChIP)-PCR analysis confirmed NR2F2 occupancy at the endogenous LCN2 promoter, with the K362R mutation exhibiting resistance to FUT2-mediated disruption of DNA binding (Fig. 4G). Consistent with these findings, qRT-PCR analysis in both KPC and PANC-1 cells revealed that NR2F2-WT significantly enhanced endogenous LCN2 transcription, while FUT2 co-expression suppressed this induction. In contrast, the NR2F2-K362R mutant completely resisted FUT2-mediated transcriptional repression, further affirming K362 as an essential regulatory ubiquitination site (Fig. 4H).

To establish clinical relevance, we conducted immunohistochemical analysis of NR2F2 in PDAC patient samples stratified by FUT2 expression. NR2F2 expression inversely correlated with FUT2 levels, being markedly elevated in samples expressing low FUT2 (Fig. 4I).

### FUT2 promotes NR2F2 degradation by facilitating its interaction with the E3 ubiquitin ligase FBXO2

**Fig. 5:**
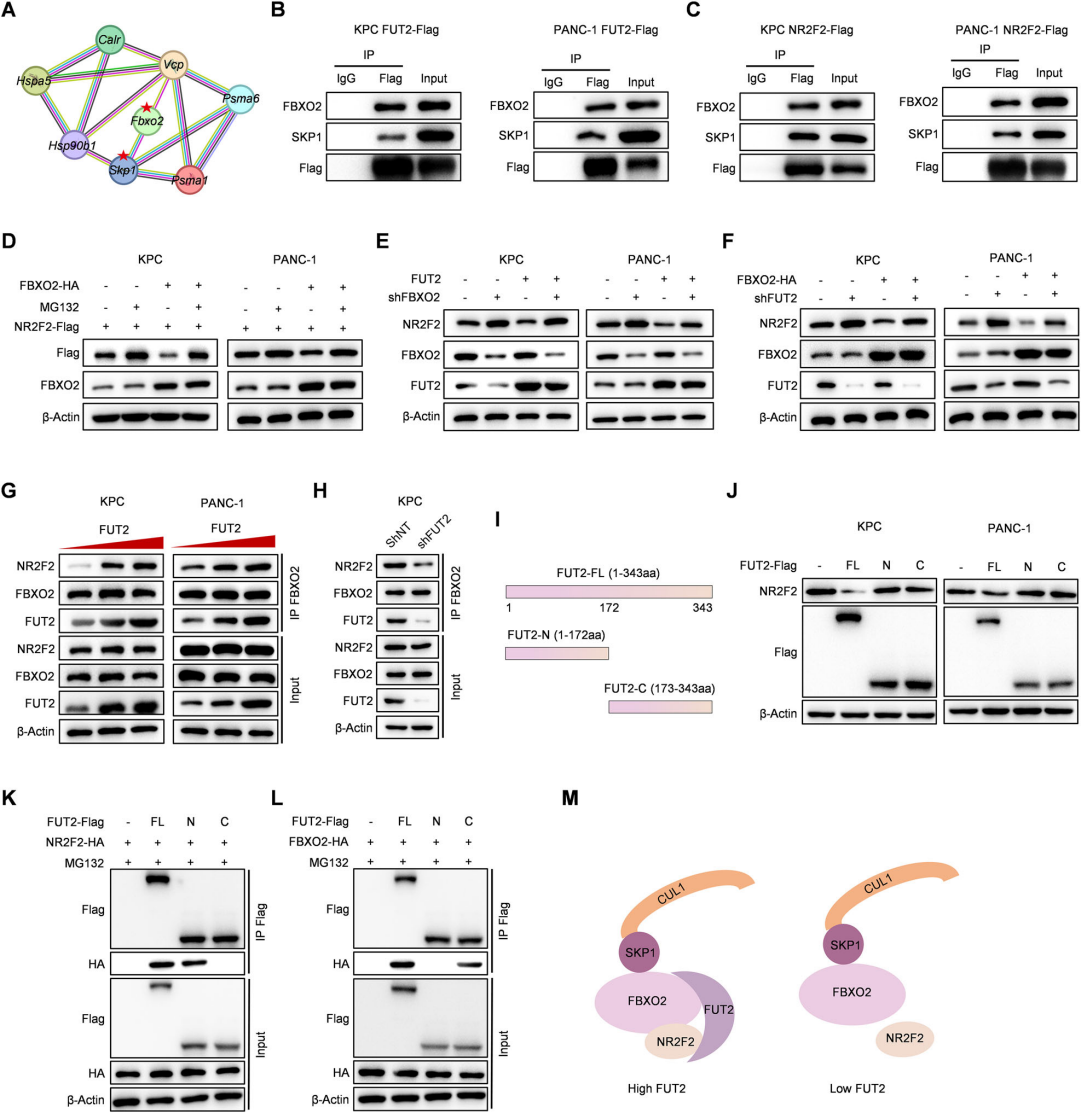
FUT2 promotes NR2F2 degradation by facilitating its interaction with the E3 ubiquitin ligase FBXO2. **A** Flag-FUT2 associated proteins were identified and analyzed using LC‒MS/MS. The Co-IP assays were performed with indicated antibodies to detect the association of FUT2 (**B**) or NR2F2 (**C**) with FBXO2 and SKP1 in KPC and PANC-1 cells. **D** The protein levels of Flag-NR2F2 were detected in KPC and PANC-1 cells with the indicated genetic manipulation and MG132 treatment. **E** NR2F2 protein levels were assessed in KPC and PANC-1 cells overexpressing FUT2-Flag or EV infected with lentivirus expressing shNT or shFBXO2. **F** NR2F2 protein levels were assessed in KPC and PANC-1 cells overexpressing FBXO2-HA or EV infected with lentivirus expressing shNT or shFUT2. **G** The endogenous interaction of FBXO2 and NR2F2 was detected in KPC and PANC-1 cells progressively overexpressing FUT2 by Co-IP assays. MG132 was added to each group. **H** The endogenous interaction of FBXO2 and NR2F2 was detected in KPC cells expressing shNT or shFUT2 by Co-IP assay. **I** Schematic diagram of the construction of full-length and truncated FUT2 proteins. **J** NR2F2 protein levels were assessed in KPC and PANC-1 cells overexpressing FUT2-FL/N/C-Flag or EV. The Co-IP assays in HEK293T cells were performed to test the interaction of NR2F2-HA (**K**) or FBXO2-HA (**L**) with FUT2-FL/N/C-Flag. **M** Schematic of FUT2-enhanced assembly of the NR2F2-FUT2-SCF^FBXO2^ holocomplex.

Given that FUT2 lacks structural domains required for direct E3 ligase activity, we hypothesized that FUT2 might serve as a scaffolding molecule to enhance interactions between NR2F2 and a specific E3 ubiquitin ligase. To investigate this, we analyzed proteins interacting with FUT2 by LC-MS/MS and identified the top 30 enriched candidates. Functional enrichment analysis revealed significant enrichment of the ubiquitin-mediated proteolysis pathway, notably highlighting the SCF complex components SKP1 and FBXO2 among the top-ranking candidates (Fig. 5A). Co-immunoprecipitation (Co-IP) assays confirmed interactions between FUT2 and FBXO2 or SKP1 in both KPC and PANC-1 cells (Fig. 5B). FBXO2 and SKP1 also exhibited tight association with NR2F2, as demonstrated by reciprocal Co-IP experiments (Fig. 5C).

To validate the role of FBXO2 in FUT2-mediated NR2F2 ubiquitination and degradation, we overexpressed FBXO2 in KPC and PANC-1 cells stably expressing either Flag-tagged NR2F2 WT or the ubiquitination-deficient NR2F2 K362R mutant. FBXO2 significantly promoted degradation of NR2F2 WT, which was reversible upon MG132 treatment, whereas NR2F2 K362R was largely resistant to destabilization induced by FBXO2, indicating a proteasome-dependent mechanism (Figs. 5D and S5A). Control experiments confirmed that FBXO2 did not mediate ubiquitination or degradation of FUT2 in these cell lines (Fig. S5B). Moreover, silencing FBXO2 markedly attenuated FUT2-induced downregulation of NR2F2 protein (Fig. 5E). Conversely, knockdown of FUT2 stabilized NR2F2 protein levels even under conditions of FBXO2 overexpression (Fig. 5F).

To further elucidate the mechanism, we co-transfected HEK293T cells with Flag-tagged NR2F2 and HA-tagged FBXO2, with or without FUT2. The results demonstrated that FUT2 significantly enhanced the interaction between FBXO2 and NR2F2 (Fig. S5C). Gradual FUT2 overexpression in KPC and PANC-1 cells proportionally increased the association between FBXO2 and NR2F2, reinforcing FUT2’s role in mediating this interaction (Fig. 5G). Conversely, FUT2 depletion significantly diminished the FBXO2-NR2F2 interaction (Fig. 5H). Collectively, these findings indicate that FUT2 promotes NR2F2 degradation by facilitating NR2F2 binding to the E3 ligase FBXO2.

To further define the interacting domains of FUT2, we generated two truncated Flag-tagged FUT2 constructs: the N-terminal fragment (FUT2-N; amino acids 1-172) and the C-terminal fragment (FUT2-C; amino acids 173-343), along with full-length FUT2 (FUT2-FL; amino acids 1-343) (Fig. 5I). Expression of these constructs in KPC and PANC-1 cells demonstrated that only FUT2-FL significantly reduced NR2F2 protein levels, whereas neither FUT2-N nor FUT2-C alone affected NR2F2 abundance (Fig. 5J). Co-IP experiments in HEK293T cells further revealed domain-specific interactions: NR2F2 interacted with both FUT2-FL and FUT2-N but not FUT2-C, whereas FBXO2 interacted with FUT2-FL and FUT2-C but not FUT2-N (Fig. 5K, L). Via in vitro binding assay, GST pulldown showed that FUT2 directly interacted with NR2F2 and FBXO2 domain specifically (Fig. S5D, E). These data suggest that FUT2 simultaneously binds FBXO2 and NR2F2 directly through distinct domains, facilitating their assembly into a stable complex essential for NR2F2 degradation (Fig. 5M).

### METTL14-mediated m6A modification represses FUT2 expression following radiotherapy

**Fig. 6:**
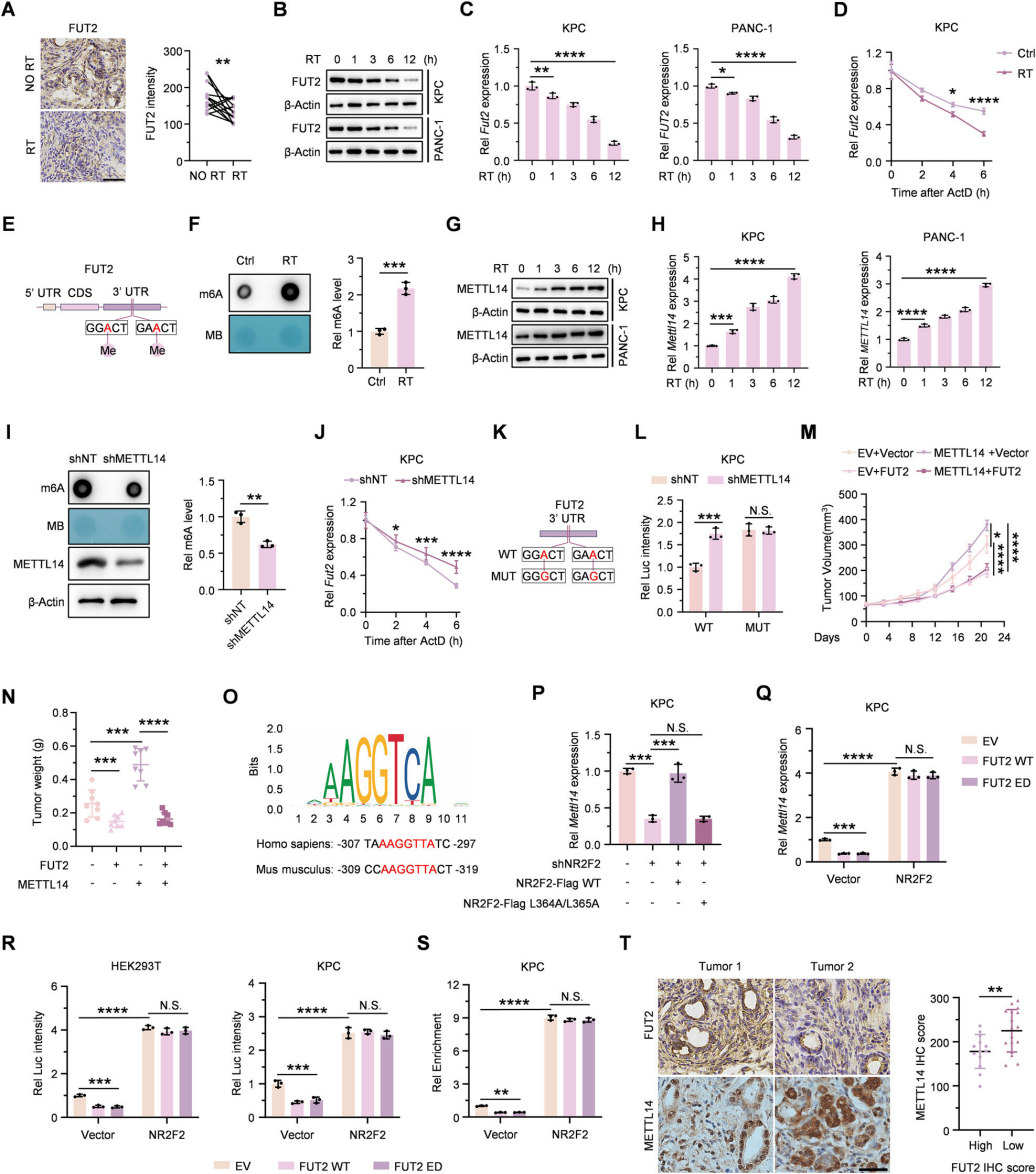
METTL14-mediated m6A modification represses FUT2 expression following radiotherapy. **A** IHC analysis of FUT2 expression was performed on paired tumor samples from 13 PDAC patients before and after radiotherapy. **B**, **C** Immunoblotting and qPCR analyses of the FUT2 expression in KPC and PANC-1 cells treated with RT for the indicated time. **D** The decay rates of *Fut2* mRNA were determined at 0, 2, 4, 6 h after treating with actinomycin D (ActD, 5ug/ml) in KPC cells with or without RT treatment. **E** The potential m6A methylation sites in the *Fut2* mRNA 3’UTR from bioinformatics prediction are shown. **F** Dot blot analysis of m6A levels in total *Fut2* RNA from irradiated and control KPC cells at 3 h post-RT. The intensity of the dot blot represents the level of m6A modification (up), and methylene blue staining was used to detect sample loading (down). **G**, **H** Immunoblotting and qPCR analyses of the METTL14 expression in KPC and PANC-1 cells treated with RT for the indicated time. **I** Dot blot analyses of total *Fut2* RNA m6A levels in irradiated KPC cells expressing shNT or shMETTL14 at 3 h post-RT. **J** The decay rates of *Fut2* mRNA were determined at 0, 2, 4, 6 h after treating with ActD (5ug/ml) in irradiated KPC cells expressing shNT or shMETTL14. **K** A luciferase reporter gene containing wild-type or mutant (A-to-G mutation) FUT2 was created for subsequent assays. **L** Transcript levels of wild-type and mutant FUT2 in KPC cells expressing shNT or shMETTL14 were detected by dual-luciferase assays. **M**, **N** EV- or METTL14- overexpressing KPC cells transfected with either Vector or FUT2 were injected subcutaneously into C57BL/6 mice, and the mice were then treated with RT at 14th day. Tumor volume (**M**), Tumor weight (**N**) are shown. **O** Screening of the METTL14 promoter region identified one of the potential NR2F2 binding motif sites in both humans and mice. **P**, **Q** Expression levels of *Mettl14* mRNA were measured in KPC cells with the indicated genetic manipulation. **R** Dual-luciferase assay was performed to measure METTL14 promoter activity in HEK293T and KPC cells co-transfected with NR2F2 and either FUT2 WT or ED. **S** ChIP-qPCR validation of METTL14 in KPC cells overexpressing EV, FUT2 WT or ED following transfection with Vector or NR2F2 and immunoprecipitated with anti-NR2F2 antibody. IgG was used as a blank control. **T** IHC staining for METTL14 was obtained and analyzed between two groups divided by IHC score of FUT2 in PDAC tissues. Scale bar, 50 μm. ^*^p < 0.05; ^**^p < 0.01; ^***^p < 0.001; ^****^p < 0.0001; N.S., not significant; Two tailed Student’s *t* test [(**A**), (**F**), (**I**) and (**T**)] or two-way ANOVA [(**C**, **D**), (**H**), (**J**), (**M**, **N**) and (**P**–**S**)].

To delineate the contribution of FUT2 expression on radioresistance, immunohistochemical analysis of paired tumor samples from PDAC patients who received preoperative radiotherapy revealed decreased FUT2 protein expression post-treatment (Fig. 6A). Consistently, we found that both FUT2 protein and mRNA levels in KPC and PANC-1 cells continually decreased in a time-dependent manner after RT treatment (Fig. 6B, C). To investigate whether irradiation-induced reductions in FUT2 mRNA resulted from impaired transcription or enhanced RNA degradation, we treated irradiated and non-irradiated cells with the transcriptional inhibitor actinomycin D (ActD). The results indicated significantly accelerated degradation of FUT2 mRNA after RT (Figs. 6D and S6A), suggesting a post-transcriptional mechanism involving reduced FUT2 mRNA stability.

Given that N6-methyladenosine (m6A) modification frequently regulates gene expression by modulating mRNA stability and translation [28], we examined potential m6A modification sites within FUT2 transcripts. Bioinformatics analysis predicted two putative m6A motifs in the 3’ UTR region of FUT2 mRNA (Fig. 6E). Dot blot assays confirmed enhanced m6A modification of FUT2 transcripts following RT relative to untreated controls (Fig. 6F), strongly suggesting RT-induced m6A-mediated degradation of FUT2 mRNA, subsequently lowering FUT2 protein levels.

To identify the m6A regulators potentially responsible for FUT2 modulation, we queried the RM2target database and identified a predicted interaction between FUT2 transcripts and the m6A methyltransferase METTL14 [29]. Time-course analysis revealed that RT induced time-dependent upregulation of METTL14 at both mRNA and protein levels in KPC and PANC-1 cells (Fig. 6G, H). Critically, evaluation of other core m6A methyltransferases including METTL3, METTL16, WTAP and VIRMA, demonstrated complete absence of RT-induced alterations (Fig. S6B), indicating METTL14-specific induction. To validate METTL14’s regulatory role, we performed dot blot assays following METTL14 silencing and RT treatment. METTL14 knockdown significantly reduced RT-induced m6A modification of FUT2 transcripts (Fig. 6I) and attenuated FUT2 mRNA degradation (Figs. 6J and S6C). To further substantiate direct m6A-dependent regulation by METTL14, we generated luciferase reporter constructs containing either wild-type (WT) or m6A-site mutant (MUT) FUT2 sequences, wherein adenosines in the predicted m6A consensus sequences were replaced by guanosines (Fig. 6K). Luciferase activity driven by FUT2-WT was significantly increased following METTL14 depletion, while FUT2-MUT reporter activity remained unaffected (Figs. 6L and S6D). These data confirm that METTL14 directly mediates FUT2 repression via site-specific m6A modification.

To explore the functional consequences of METTL14-mediated FUT2 regulation on RT response, we generated stable cell lines overexpressing METTL14 alone or in combination with FUT2, implanted these cells into C57BL/6 mice, and administered RT on day 14 post implantation. Notably, the radioresistant phenotype conferred by METTL14 overexpression was effectively reversed by co-expression of FUT2, resulting in tumor suppression comparable to FUT2 overexpression alone (Figs. 6M, N, and S6E). Consistent with our earlier findings indicating that FUT2 enhances radiosensitivity primarily through increased apoptosis rather than inhibition of proliferation, Ki67 and TUNEL staining demonstrated similar patterns (Fig. S6F).

Surprisingly, bioinformatics analysis via the JASPAR database revealed a high-confidence NR2F2-binding motif in the METTL14 promoter region that is highly conserved in humans and mice (Fig. 6O). Quantitative RT-PCR analysis in both KPC and PANC-1 cells revealed that NR2F2 depletion significantly suppressed METTL14 transcription, but this decrease was restored after reintroducing NR2F2-WT but not NR2F2-L364A/L365A mutant, underscoring NR2F2’s transcriptional activity as essential for METTL14 induction (Figs. 6P and S6G). Complementary gain-of-function studies demonstrated that NR2F2 overexpression enhanced METTL14 transcription. Critically, in FUT2 WT- or ED-overexpressing cells, reconstitution of NR2F2 fully abrogated FUT2-mediated METTL14 suppression, restoring expression to levels comparable to NR2F2 overexpression alone (Figs. 6Q and S6H). Luciferase reporter assays demonstrated that NR2F2 overexpression robustly activated METTL14 promoter activity and restored the inhibitory effect of overexpression of FUT2 WT or ED on METTL14 (Fig. 6R). Consistently, chromatin immunoprecipitation with an anti-NR2F2 antibody followed by quantitative PCR analysis confirmed NR2F2 occupancy at the endogenous METTL14 promoter, with the NR2F2 overexpression exhibiting resistance to FUT2-mediated disruption of DNA binding (Fig. 6S). Collectively, these data demonstrate that elevated NR2F2 due to FUT2 reduction transcriptionally upregulates METTL14, establishing a feedforward loop that reinforces FUT2 suppression.

Finally, immunohistochemical analysis of PDAC patient samples revealed an inverse correlation between METTL14 and FUT2 protein expression (Fig. 6T), further underscoring the clinical relevance of this regulatory axis.

## Discussion

**Fig. 7:**
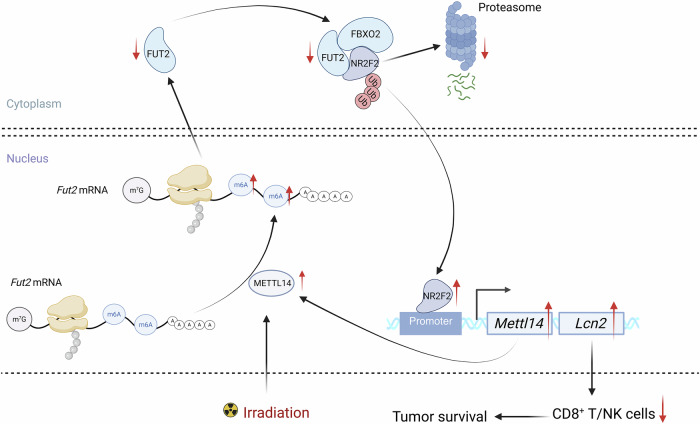
Schematic model of the mechanism by which FUT2-mediated LCN2 expression determines the efficiency of radiation therapy.

In this study, we demonstrated that FUT2 suppresses tumor immune evasion in pancreatic cancer independently of its canonical enzymatic activity. Specifically, FUT2 protein abundance critically determines radiosensitivity in pancreatic ductal adenocarcinoma (PDAC). Mechanistically, RT induces upregulation of the m6A methyltransferase METTL14, which catalyzes m6A methylation of FUT2 mRNA, resulting in accelerated mRNA degradation and decreased FUT2 protein levels. Reduced FUT2 expression diminishes the association between the E3 ubiquitin ligase FBXO2 and the transcription factor NR2F2, thereby stabilizing NR2F2 and promoting transcriptional activation of its downstream effector LCN2. Critically, elevated NR2F2 transcriptionally upregulates METTL14, establishing a self-reinforcing FUT2 suppression circuit. Secreted LCN2 subsequently promotes apoptosis of tumor-infiltrating cytotoxic CD8^+^ T cells and reduces cytotoxic NK cell infiltration, fostering an immunosuppressive TIME and ultimately facilitating tumor survival post-RT (Fig. 7). Importantly, we demonstrated that combining RT with an LCN2-neutralizing antibody markedly improved infiltration of CD8^+^ T cells and NK cells, substantially enhancing therapeutic efficacy.

RT primarily induces tumor cell death via direct DNA damage and the generation of cytotoxic free radicals [30]. However, therapy-induced immunosuppression within the tumor microenvironment remains a significant obstacle, reducing the efficacy and durability of radiotherapy [10]. Recent studies highlight the synergistic antitumor effects achieved by combining RT with immune checkpoint inhibitors, such as anti-PD-1/PD-L1 antibodies [31, 32]. Additionally, targeting immunosuppressive cell populations, such as using anti-CSF-1 antibodies to deplete tumor-associated macrophages (TAMs) during RT, delays pancreatic tumor progression, with enhanced efficacy when combined with PD-L1 inhibition [33]. These findings underscore the potential for improving RT outcomes by strategically modulating the TIME, aligning with our demonstration of synergistic enhancement of RT efficacy by LCN2 neutralizing antibodies.

Leveraging an orthotopic KPC pancreatic cancer model in immunocompetent mice and employing a CRISPR-Cas9 library targeting metabolic enzymes, we identified FUT2 as a critical regulator of RT response. Despite FUT2’s classical function in catalyzing α-1,2-fucosylation of glycoproteins and glycolipids [34–37], our results revealed a noncanonical, enzyme-independent role wherein FUT2 abundance alone influenced antitumor immune responses during RT. Such noncanonical roles of metabolic enzymes, influencing diverse biological processes beyond their enzymatic activity, have increasingly been recognized [38]. Our findings expand this paradigm by highlighting FUT2’s capacity to modulate immune surveillance through protein interactions rather than enzymatic catalysis.

To delineate the mechanism by which FUT2 represses LCN2 expression, we identified and validated NR2F2 as a transcription factor directly regulating LCN2. Our analysis indicated that FUT2 reduces NR2F2 protein levels through the ubiquitin-proteasome system. LC-MS/MS screening of FUT2-interacting proteins identified components of the SCF complex, notably FBXO2 and SKP1. We further established that FUT2 stabilizes the FBXO2-NR2F2 interaction via distinct structural domains, thereby facilitating NR2F2 ubiquitination and proteasomal degradation. This is the first report elucidating the molecular interplay whereby FUT2 directs FBXO2-mediated degradation of NR2F2. Given NR2F2’s established roles in promoting tumor proliferation, angiogenesis, and lymphangiogenesis [39–41], the FUT2-FBXO2-NR2F2 regulatory axis may offer a mechanistic rationale for targeting NR2F2 degradation in cancer therapy.

Importantly, our study identifies METTL14 as a crucial upstream regulator modulating this axis in response to RT. We demonstrated that RT upregulates METTL14, which in turn catalyzes m6A methylation on FUT2 mRNA, accelerating its decay and ultimately reducing FUT2 protein abundance. This reduction in FUT2 is the key event initiating the disruption of the FBXO2-NR2F2 interaction, leading to NR2F2 stabilization and LCN2 upregulation. Critically, we uncovered that elevated NR2F2 transcriptionally upregulates METTL14 expression, thereby establishing a potent feedforward loop that amplifies FUT2 suppression and consolidates the RT-induced immunosuppressive phenotype. This METTL14-FUT2-NR2F2 feedback circuit represents a novel molecular mechanism underpinning radioresistance by chronically dampening antitumor immunity through LCN2. It should be mentioned that the clinical correlations presented in this study, while statistically significant, are derived from a single-institution cohort of limited size. Thus, further validation of FUT2 as a predictive biomarker for radiotherapy response and its association with the NR2F2-LCN2 immune axis in larger, multi-institutional prospective cohorts is warranted to confirm its generalizability and clinical utility.

LCN2 is a secreted glycoprotein implicated in tumor growth, apoptosis, migration, and angiogenesis through iron-transport mechanisms [23, 42–44]. Our study indicates that tumor-derived LCN2 exacerbates immunosuppression in pancreatic cancer by inducing apoptosis in tumor-infiltrating CD8^+^ T cells and potentially influencing NK cells similarly. Critically, we demonstrate that therapeutically targeting LCN2 using neutralizing antibodies significantly enhanced intratumoral CD8^+^ T and NK cell infiltration, thereby potentiating RT efficacy in PDAC. This finding aligns with and is reinforced by prior studies showing LCN2 blockade improves therapeutic responses to sorafenib in liver cancer and gemcitabine in pancreatic cancer [45, 46]. Furthermore, evidence exists for LCN2 contributing to radioresistance via distinct mechanisms, such as promoting DNA repair and proliferation in other malignancies like nasopharyngeal carcinoma [47]. Collectively, these diverse lines of evidence, encompassing our discovery of LCN2’s novel immunosuppressive mechanism within the PDAC TIME driving radioresistance, its established roles in chemoresistance, and its contribution to radioresistance through non-immune pathways in nasopharyngeal carcinoma, strongly support the therapeutic relevance of targeting LCN2. Consequently, our demonstration of enhanced radiotherapy outcomes via LCN2-neutralizing antibodies robustly positions this combination strategy as a highly promising approach to reverse TIME-associated radioresistance in PDAC. Nevertheless, future clinical translation requires rigorous evaluation of antibody specificity and potential off-target effects on non-tumor cell populations.

In conclusion, we reveal a novel regulatory mechanism wherein FUT2 facilitates FBXO2-mediated degradation of NR2F2, subsequently repressing LCN2 transcription and enhancing antitumor immunity during RT. We further uncover METTL14-mediated m6A modification as the upstream regulatory mechanism driving FUT2 mRNA degradation in response to irradiation. Moreover, increased NR2F2 resulting from decreased FUT2 transcriptionally upregulates METTL14, establishing a feedforward loop that reinforces FUT2 suppression. Since FUT2 downregulates its mRNA levels during radiotherapy, monitoring FUT2 mRNA expression in circulating tumor cells may help predict radiotherapy efficacy. Furthermore, as a putative radiosensitizer with a relatively short protein length of only 343 amino acids, we plan to identify the specific functional domain responsible for its radiosensitizing effect. Subsequently, molecular dynamics simulations will be employed to model the complex structure of the FUT2/FBXO2 interaction region. Based on this structural insight, a truncated peptide derived from the key functional region of FUT2 will be designed and packaged into nanoparticle carriers for targeted delivery to tumor sites. FUT2 expression may thus represent a clinically relevant biomarker for predicting RT response in PDAC, and combining RT with LCN2-neutralizing antibodies emerges as a promising therapeutic strategy to improve clinical outcomes in pancreatic cancer patients.

## Materials and methods

### Cell lines and culture conditions

KPC and PANC-1 cells were donated by Professor Zhigang Zhang and human embryonic kidney (HEK) 293T cell lines was obtained from the American Type Culture Collection. All cells were cultured at 37 °C with 5% CO_2_ in Dulbecco’s modified Eagle’s medium (DMEM) supplemented with 10% fetal bovine serum (FBS), penicillin (100 U/ml) and streptomycin (100 µg/ml). All cell lines were tested to confirm no mycoplasma contamination.

### Mice

Male C57BL/6 mice aged 6 to 8 weeks, used to establish the PDAC orthotopic and subcutaneous xenograft models, were sourced from the Guangdong Medical Animal Center and maintained in a specific pathogen-free facility under standardized conditions. Groups of four to five mice were housed in individually ventilated cages, with no additional enrichment. The facility maintained a 12-h light/dark cycle, along with controlled temperature and humidity, and the mice had unrestricted access to food and water. All handling, dosing, and humane endpoints were performed by experienced experimenters to minimize animal distress. The experiments complied with the National Institutes of Health Guide for the Care and Use of Laboratory Animals and were approved by the Research Ethics Committee of Guangzhou University[NO.2025(049)].

### In vivo CRISPR knockout screening of metabolic enzymes

The in vivo CRISPR screen was carried out as previously outlined [48]. In brief, a library of 1516 mouse metabolic enzyme genes, each targeted by three sgRNAs, along with 91 nontargeting sgRNAs, was cloned into the LentiCRISPR-v2 vector. Lentiviruses were produced in 293T cells by co-transfecting the pooled library, psPAX2, and pMD2.G at a 4:3:1 ratio (m/m/m). Polyethyleneimine (PEI) was used as the transfection reagent in a 4:1 (m/m) ratio relative to the plasmids. After lentivirus packaging, concentration, and titer determination, luciferase-expressing target cells were infected at an MOI of 0.3, ensuring a coverage of over 500 cells per sgRNA. Following 4 days of puromycin selection, positive cells were collected for orthotopic transplantation into the mouse pancreas, with six mice per group. Thirteen days post-transplantation, the mice were treated with radiotherapy. Three days later, pancreatic tumors were harvested, and total genomic DNA was extracted for the amplification of sgRNA fragments. The amplified products were then subjected to next-generation sequencing using an Illumina sequencer, and the abundance of each sgRNA was quantified.

### Mouse tumor model and treatments

A mouse pancreatic tumor orthotropic transplantation model was established using the mouse PDAC cell line KPC. Briefly, C57BL/6 mice between the ages of 6 and 8 weeks were anesthetized by isoflurane and placed on an experimental pad in a lateral position to raise the upper left side of the abdomen. Using sterile surgical instruments make a 1 cm transverse incision and gently remove the spleen from the abdominal cavity. Then exposing and locating the tail of the pancreas adjacent to the spleen by a sterile cotton bud. Using a insulin syringe, the pancreas was injected with KPC cells (1 × 10^6^ cells in 50 µl PBS). Returning the spleen and pancreas to the abdominal cavity and closing the incision with an absorbable suture. After surgery, mice were incubated at 37 °C until they awakened. A subcutaneous tumor transplantation model was established using KPC cells. A total of 1 × 10^6^ cells were injected subcutaneously into 6- to 8-week-old C57BL/6 mice. Tumor dimensions were recorded every 3 days using micro-calipers, and volumes were calculated based on length (*L*) and width (*W*) using the formula *LW*^2^/2. The investigator who performed the measurements was blinded to experimental group allocation. At the experimental endpoint, the mice were euthanized, and the tumors were dissected carefully.

Irradiation was performed using an RS2000 x-ray linear accelerator (RAD SOURCE, Buford, GA, USA) at 160 kV, 25 mA with 0.3 mm Cu filter. Prior to the radiation, mice were positioned in the prone orientation, and a CT scan or bioluminescence was employed to mark the target area in the corresponding skin. Radiation was delivered at a dose rate of 1.338 Gy/min. A single 8 Gy dose of radiation was delivered to tumor area, whereas the tumor-surrounding tissue was protected by using the high-throughput local irradiator. LCN2 recombinant protein or antibody was executed at Day 14 by intratumoral or intravenous injection respectively, as shown in the related figure. The doses were 10 µg per mouse for LCN2 recombinant protein and 4 mg/kg for LCN2-neutralizing antibody (twice a week, Cat# MAB18571, clone: 228418, R&D Systems) or isotype control antibody (Cat# MAB006, clone: 54447, R&D Systems).

### Tumor-infiltrating immune cell isolation and flow cytometry

Tumor tissues were isolated and digested using the Singleron Python tissue dissociator with pancreatic tissue single-cell dissociation solution (singleron) at 37 °C for 15 min. The resulting cell suspension was filtered through a 40-micron strainer and centrifuged at 350 × *g* for 4 min at 4 °C. The cell pellet was resuspended in 10 ml percoll (37.5%) in a 15 mL tube. The supernatant was discarded after centrifugation at 600 × *g* for 30 min at 22 °C, and the cell pellet was collected. One milliliter of erythrocyte lysis solution was added to the cell pellet, mixed thoroughly by pipetting, and incubated on ice for 2–3 min to lyse erythrocytes. The lysis was stopped by adding 9 mL of PBS, followed by centrifugation at 350 × *g* for 5 min, after which the cell pellet was collected. The cell pellet was resuspended in 250 µL of PBS supplemented with 2% FBS for antibody staining. To block nonspecific binding, single-cell suspensions were incubated with anti-mouse CD16/CD32 antibodies (Thermo Fisher) for 15 min. Fluorophore-conjugated antibodies were then added and allowed to stain on ice in the dark for 30 min. Isotype controls were used to verify the specificity of the primary antibodies. The flow cytometry antibodies used in this study were anti-mouse CD45- PE eFluor 610 (1:200, Invitrogen, #61-04510-82), anti-mouse CD3-APC (1:200, Biolegend, #100312), anti-mouse CD8-Alexa Fluor 700 (1:200, eBioscience, #56-0081-82), and anti-mouse NK1.1-APC eFluor 780 (1:200, Biolegend, #47-5941-82). Flow cytometry analysis was conducted on a BD FACSAria™ III system, and data were subsequently analyzed using FlowJo software (v10.4.2).

### Apoptosis analysis

CD8^+^ T cells were isolated and purified from mouse spleens using the Mouse CD8^+^ T Cell Isolation Kit. The cells were treated with or without recombinant LCN2 protein (low dose: 10 nM; high dose: 100 nM) for 12 h. Following treatment, the cells were harvested, washed with PBS, and stained with fluorescein isothiocyanate (FITC)-conjugated Annexin V using the Annexin V-FITC/PI apoptosis detection kit (Vazyme) in accordance with the manufacturer’s protocol. The apoptosis levels were subsequently analyzed by flow cytometry.

### Single-cell RNA sequencing

The single-cell suspensions were prepared following the methods and procedures described above and then loaded onto a microwell chip using the Singleron Matrix® Single-Cell Processing System. Barcoding beads were collected, and mRNA was reverse transcribed into cDNA, followed by PCR amplification. The amplified cDNA was fragmented, adapter-ligated, and used to construct libraries with the GEXSCOPE® Single-Cell RNA Library Kit (Singleron). The libraries were then pooled and sequenced on an Illumina NovaSeq 6000 platform. Raw sequencing data were processed using CeleScope to generate gene expression matrices for downstream analysis, including dimensionality reduction, clustering, and differential expression analysis.

### RNA sequencing and analysis

Total RNA was extracted for RNA sequencing on an Illumina HiSeq 2500 platform. Sequencing data were analyzed and managed using the BaseSpace Sequence Hub. A list of differentially expressed genes (DEGs) was obtained based on a *p*-value < 0.05 and a fold change >1.5 or <0.65.

### Mass spectrometry analysis

To identify the interacting proteins of FUT2, Flag-FUT2 was immunoprecipitated from KPC cells using anti-Flag resin beads. The precipitated complexes were boiled with 40 μl 1× loading buffer at 95 °C for 10 min and analyzed by mass spectrometry. To identify the ubiquitination sites of NR2F2, NR2F2-FLAG was immunoprecipitated after co-expression with Ub-HA. The ubiquitination sites on NR2F2 were then identified and analyzed by mass spectrometry. For differential proteomic analysis, KPC cells overexpressing FUT2 or EV were subjected to irradiation with 6 Gy and mass spectrometry.

### Immunohistochemistry (IHC)

Paraffin-embedded tissue sections from human PDAC specimens were subjected to immunohistochemical (IHC) staining with antibodies targeting FUT2 or LCN2. The IHC scores were assessed by two independent authors who were blinded to the patients’ clinicopathological information. Staining intensity was graded on a scale of 0 to 3: 0, negative; 1, weak; 2, moderate; and 3, strong. We assigned the proportion of stained tumor cells was indicated as a percentage (0 ≤ X ≤ 100). The score (H-score) was calculated using the following formula: 3× the percentage of strongly stained signal + 2× the percentage of moderately stained signal + 1× the percentage of weakly stained signal, yielding a total range of 0 to 300. The staining intensity and proportion scores were combined to derive a total score following previously established methods. Subcutaneous tumor tissues were stained with antibodies against Ki67 and with a TUNEL BrightRed Apoptosis Detection kit.

### Immunofluorescence (IF)

The tissue sections of human PDAC specimens were co-incubated with primary antibodies against CD8a and FUT2 or LCN2, secondary antibodies conjugated with Alexa 488 or Alexa 555 and 4’,6-diamidino-2-phenylindole (DAPI) according to the manufacturer’s instructions. Images were captured using Zeiss LSM 900 laser confocal microscope equipped with Airyscan. The IF scores of FUT2 and LCN2 were evaluated using the same criteria as the IHC analysis. Scores were compared with the number of CD8+ T cells and analyzed by calculating the Pearson correlation coefficient.

### Co-immunoprecipitation (Co-IP)

Anti-Flag M2 magnetic beads was incubated with Flag-tagged cell lysates at 4 °C for 4 h. A portion of the lysate was reserved as input. The beads were then washed with protein lysis buffer and resuspended in 40 μl of 1× loading buffer. The purified Flag-tagged proteins and their associated complexes were boiled at 95 °C for 5 min. The proteins were subsequently analyzed by immunoblotting using the specified antibodies. To perform immunoprecipitation (IP) of endogenous proteins, Protein A/G Magnetic Beads were used according to the manufacturer’s instructions. After incubation, the beads were washed and the samples were eluted by resuspending the beads in 40 μl of 1× loading buffer, followed by heating at 95 °C for 5 min. The eluted proteins were then analyzed using immunoblotting.

### Statistical analysis

All statistical analyses were conducted using GraphPad Prism software (v.8.0). Each experiment was independently repeated at least three times, with results presented as the means ± SD or the means ± SEM. Comparisons between two independent groups were performed using an unpaired two-tailed Student’s *t*-test, while comparisons among more than two groups were assessed using one-way or two-way analysis of variance (ANOVA) followed by Bonferroni correction. *P* value < 0.05 was considered statistically significant.

## Supplementary information

Supplementary Information

Western Blotting Original Image

## Data Availability

Data supporting the findings of this study are available from the corresponding authors upon reasonable request. The mass spectrometry proteomics data have been deposited to the ProteomeXchange Consortium via the iProX partner repository with the dataset identifier PXD062766. The raw sequencing data generated through single-cell RNA sequencing (scRNA- sequencing) and single-guide RNA sequencing (sgRNA-sequencing) are available in the BioProject database with the accession BioProject: PRJNA1248070, and BioProject: PRJNA1247468.
