# Supplementary material for: FUT2 enhances anti-tumor immunity in pancreatic cancer radiotherapy by driving FBXO2-mediated degradation of NR2F2
Source: Cell Death Dis. 2025 Dec 23;17(1):126. doi: 10.1038/s41419-025-08378-2 (PMC12848027; doi:10.1038/s41419-025-08378-2)
Supplement: Supplementary file 1 — Supplementary Information [file 41419_2025_8378_MOESM1_ESM.docx]

**Supplemental Information**

**Supplemental Figures and Legends**


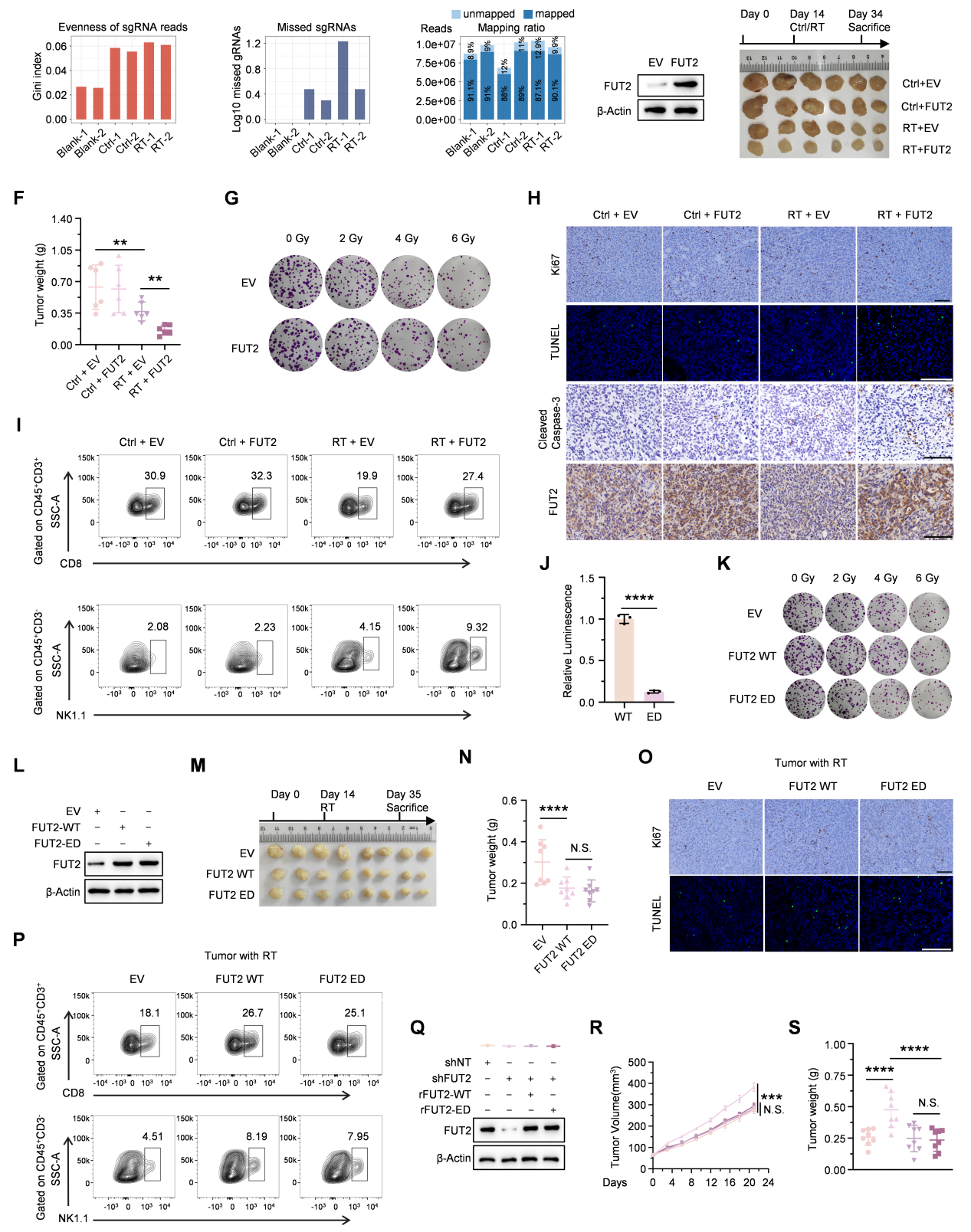


**Fig. S1 FUT2 synergistically enhances pancreatic cancer cell death following radiotherapy.** (**A** to **C**) Quality check of evenness of sgRNA reads (A), missed sgRNAs (B) and mapping ratio (C) of the in vivo CRISPR screening data. (**D**) Overexpression of FUT2 in KPC cells was confirmed by immunoblotting analysis. (**E** and **F**) Subcutaneous transplantation of EV- or FUT2-overexpressing KPC cells into C57BL/6 mice and the mice were then treated with or without RT at 14th day. Tumor images are shown (E). Tumor weight was measured (F). (**G**) Representative images of colony formation of KPC cells overexpressing EV or FUT2 following exposure to varying doses of RT. (**H** and **I**) Subcutaneous transplantation of EV- or FUT2-overexpressing KPC cells into C57BL/6 mice and the mice were then treated with or without RT at 14th day. Representative images of Ki67, TUNEL, cleaved caspase-3 and FUT2 staining of tumor (H) and flow cytometry (I) are shown. Scale bar, 100 μm. (**J**) Enzymatically dead FUT2 (FUT2-ED) was constructed by introducing point mutations V206G, T288N and A300T into wild-type FUT2 (FUT2-WT). The relative enzyme activity of FUT2 ED was measured compared with WT in KPC cells. (**K**) Representative images of colony formation of KPC cells overexpressing EV, FUT2 WT, or ED following exposure to varying doses of RT. (**L**) Overexpression of FUT2 WT or ED in KPC cells was confirmed by immunoblotting analysis. (**M** to **P**) Subcutaneous transplantation of EV-, FUT2 WT-, or ED-overexpressing KPC cells into C57BL/6 mice and the mice were then treated with RT at 14th day. Tumor images (M), measurement of tumor weight (N), representative images of Ki67 and TUNEL staining (O), representative images of flow cytometry (P) are shown. Scale bar, 100 μm. (**Q** to **S**) KPC cells expressing shNT, shFUT2, and shFUT2 rescued with rFUT2 WT or ED were subcutaneously injected to C57BL/6 mice that were then treated with RT at 14th day. Immunoblotting analyses of indicated proteins (Q). Tumor volumes (R) and tumor weight (S) were measured. **p* < 0.05; ***p* < 0.01; ****p* < 0.001; *****p* < 0.0001; N.S., not significant; two-way ANOVA [(**F**), (**N**), (**R**) and (**S**)] or Two tailed Student’s t test (**J**).


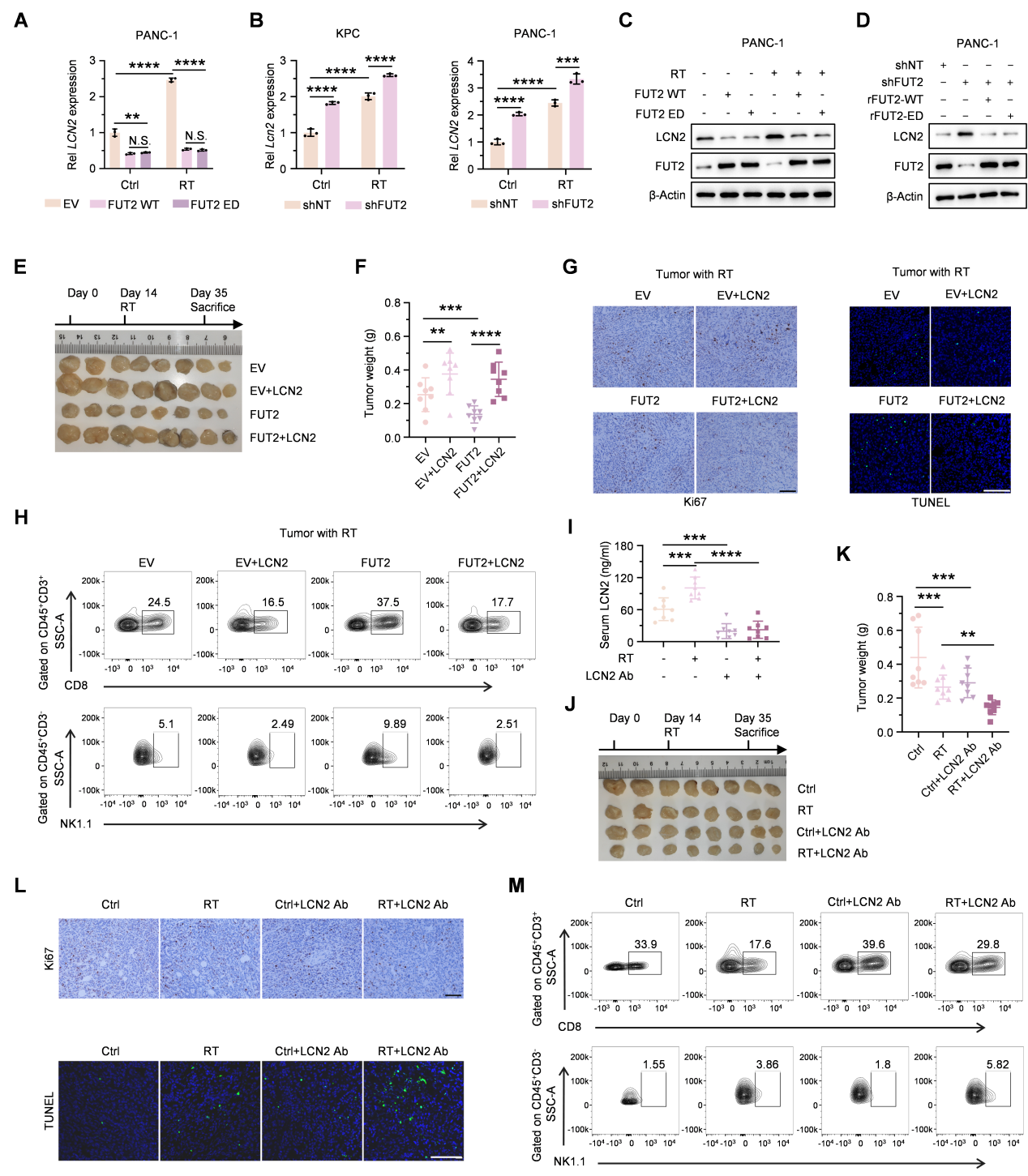


**Fig. S2 FUT2 enhances radiotherapy efficacy by suppressing LCN2 expression.** (**A**) Expression levels of LCN2 mRNA were measured in PANC-1 cells overexpressing EV, FUT2 WT, or ED in the absence or presence of RT, with samples collected 12 hours post-RT. (**B**) Expression levels of LCN2 mRNA were measured in KPC and PANC-1 cells expressing shNT or shFUT2 in the absence or presence of RT, with samples collected 12 hours post-RT. (**C**) Immunoblotting analyses of indicated proteins in PANC-1 cells overexpressing EV, FUT2 WT, or ED at 24 hours post-RT. (**D**) Immunoblotting analyses of indicated proteins in PANC-1 cells expressing shNT, shFUT2, and shFUT2 rescued with rFUT2 WT or ED. (**E** to **H**) Subcutaneous transplantation of EV or FUT2-overexpressing KPC cells into C57BL/6 mice that were then treated with RT at 14th day. Exogenous LCN2 supplementation was performed as described in the supplementary methods. Tumor images (E), Tumor weight (F), representative images of Ki67 and TUNEL staining (G), representative images of flow cytometry (H) are shown. Scale bar, 100 μm. (**I** to **M**) KPC cells were subcutaneously transplanted into C57BL/6 mice that were then treated with RT or LCN2 neutralizing antibodies as described in the supplementary methods. ELISA of secretory LCN2 in the tumor tissues (I), Tumor images (J), Tumor weight (K), representative images of Ki67 and TUNEL staining (L), representative images of flow cytometry (M) are shown. Scale bar, 100 μm. ***p* < 0.01; ****p* < 0.001; *****p* < 0.0001; N.S., not significant; two-way ANOVA [(**A**), (**B**), (**F**), (**I**) and (**K**)].


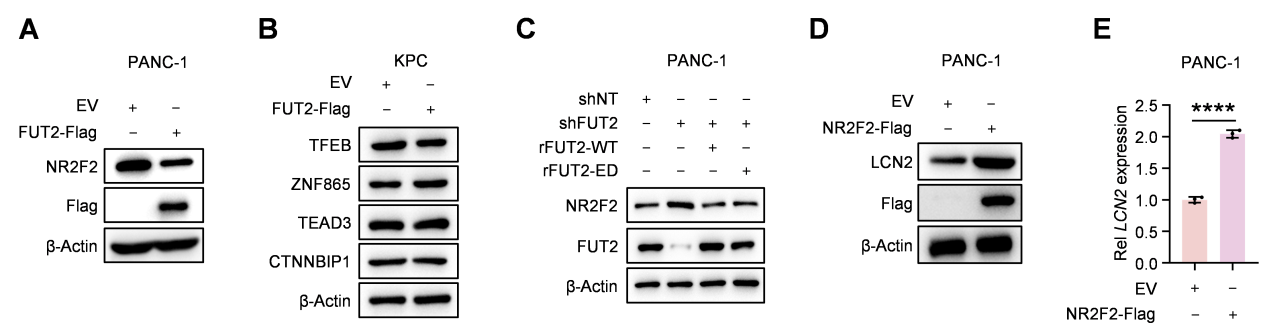


**Fig. S3 FUT2 represses LCN2 transcription by facilitating NR2F2 destabilization.** (**A** to **C**) Immunoblotting analyses of indicated proteins in KPC or PANC-1 cells with the indicated genetic manipulation. (**D** and **E**) Expression levels of LCN2 protein (D) and mRNA (E) were measured in PANC-1 cells overexpressing EV or NR2F2-Flag. ****P < 0.0001; Two tailed Student’s t test (**E**).


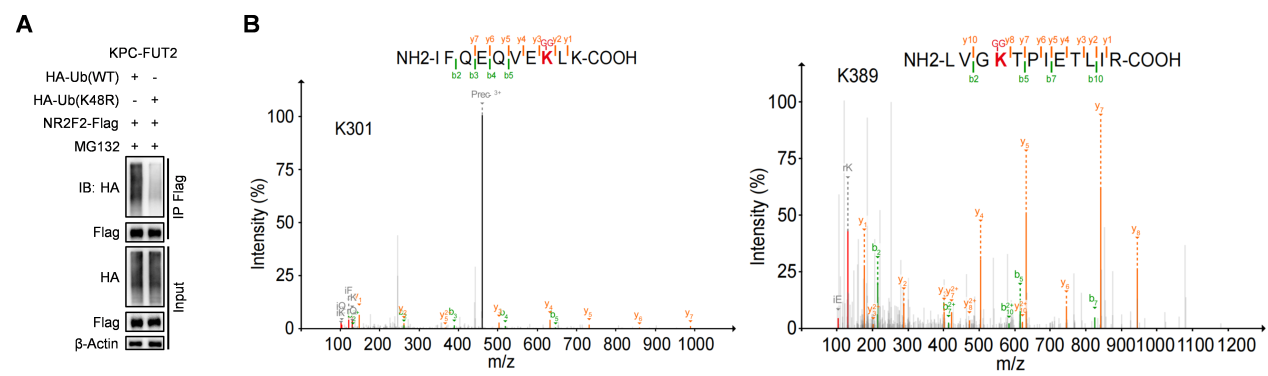


**Fig. S4 FUT2 promotes NR2F2 degradation through ubiquitination at lysine 362 via the proteasomal pathway.** (**A**) Ubiquitination assays of NR2F2 in FUT2-overexpressing KPC cells transfected with NR2F2-Flag and HA-ub (WT or K48R). (**B**) NR2F2-Flag was subjected to LC‒MS and identified three potential ubiquitination sites.


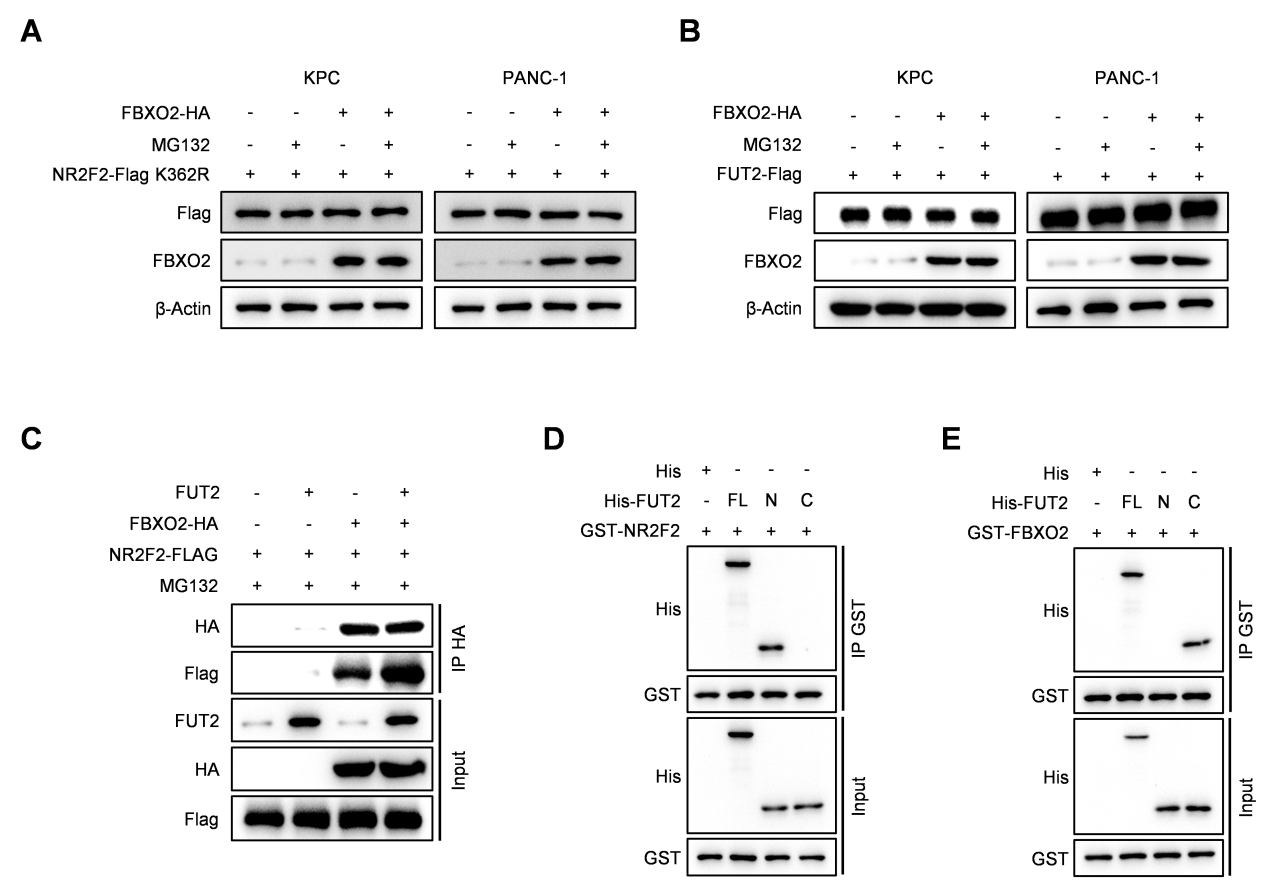


**Fig. S5 FUT2 promotes NR2F2 degradation by facilitating its interaction with the E3 ubiquitin ligase FBXO2. (A) The protein levels of Flag-NR2F2 K362R were detected in KPC and PANC-1 cells with the indicated genetic manipulation and MG132 treatment.** (**B**) The protein levels of Flag-FUT2 were detected in KPC and PANC-1 cells with the indicated genetic manipulation and MG132 treatment. (**C**) The Co-IP assay in HEK293T cells was performed to test the interaction of FBXO2-HA with NR2F2-Flag in the absence or presence of FUT2 overexpression. (**D** and **E**) In vitro pulldown and immunoblotting assays were performed with indicated proteins to test the direct interaction of NR2F2 (D) or FBXO2 (E) with FUT2-FL/N/C. GST-tagged NR2F2 and His-tagged FUT2-FL/N/C were purified from *E.coli* and incubated together.


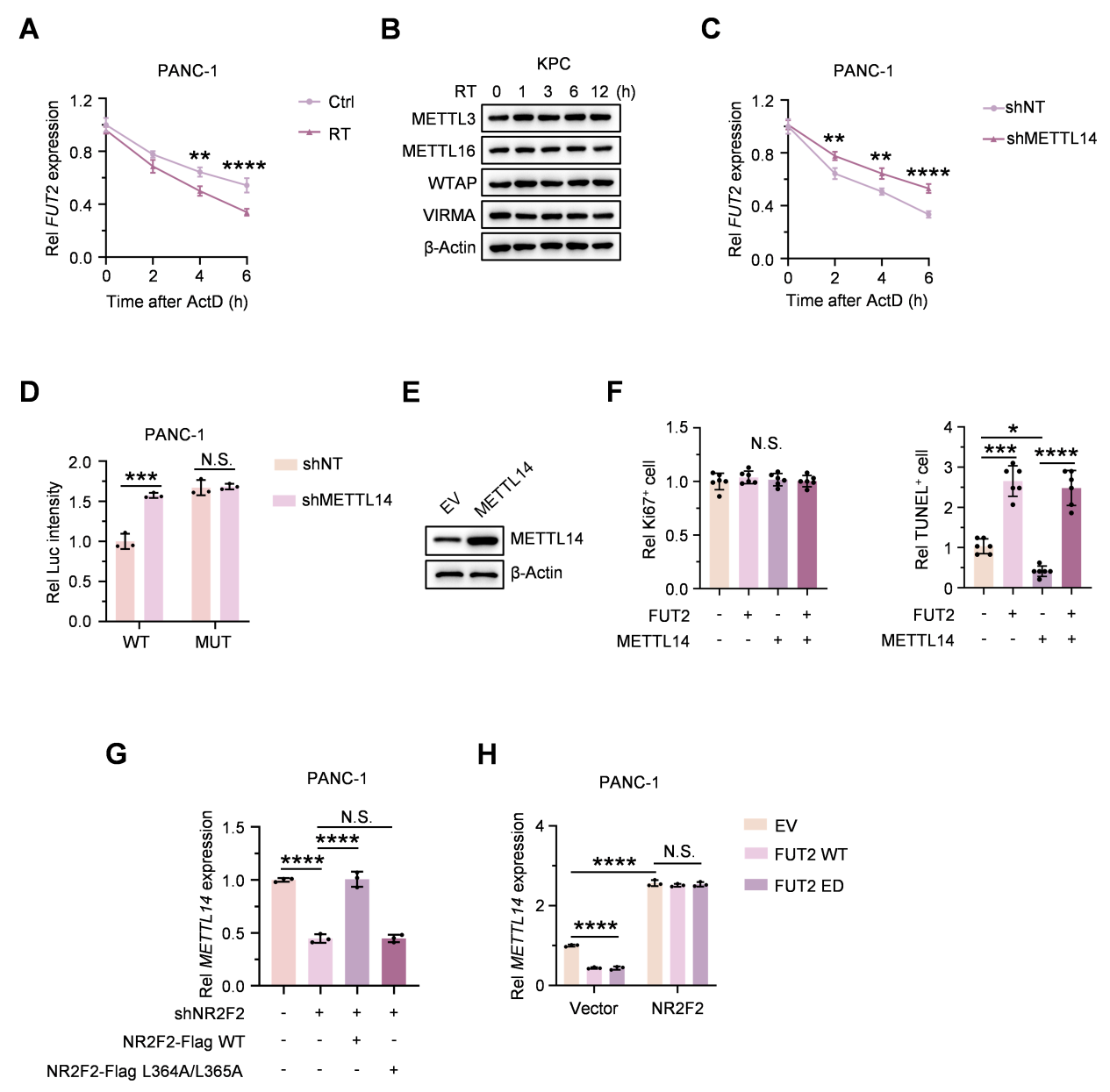


**Fig. S6 METTL14-mediated m6A modification represses FUT2 expression following radiotherapy.** (**A**) The decay rates of FUT2 mRNA were determined at 0, 2, 4, 6h after treating with actinomycin D (ActD, 5ug/ml) in PANC-1 cells with or without RT treatment. (**B**) Immunoblotting analyses of indicated proteins in cells treated with RT for the indicated time. (**C**) The decay rates of FUT2 mRNA were determined at 0, 2, 4, 6h after treating with ActD (5ug/ml) in irradiated PANC-1 cells expressing shNT or shMETTL14. (**D**) Transcript levels of wild-type and mutant FUT2 in PANC-1 cells expressing shNT or shMETTL14 were detected by dual-luciferase assays. (**E**) Overexpression of METTL14 in KPC cells was confirmed by immunoblotting analyses. (**F**) EV- or METTL14- overexpressing KPC cells transfected with either Vector or FUT2 were injected subcutaneously into C57BL/6 mice, and the mice were then treated with RT at 14th day. Quantifications of tumor proliferative and dead index determined by Ki67 and TUNEL staining were shown. (**G** and **H**) Expression levels of METTL14 mRNA were measured in PANC-1 cells with the indicated genetic manipulation. *p < 0.05; **p < 0.01; ***p < 0.001; ****p < 0.0001; N.S., not significant; two-way ANOVA [(**A**), (**C**), (**D**) and (**F**-**H**)].

**Plasmids construction and transfection**

PCR-amplified mouse FUT2, NR2F2, FBXO2, LCN2, METTL14 were cloned into pCDH-SFB-Flag, pCDH-hygro, pCDH-HA or pET28a-His/GST vectors. FUT2-V206G/T288N/A300T, NR2F2-L364A/L365A, NR2F2-K301R, -K362R, -K389R and FUT 3’ UTR-MUT were generated using a QuikChange sitedirected mutagenesis kit (Stratagene, La Jolla, CA, USA). HA-ub, HA-K6R-ub, HA-K11R-ub, HA-K27R-ub, HA-K29R-ub, HA-K33R-ub, HA-K48R-ub or HA-K63R-ub were obtained from The Tenth People's Hospital of Shanghai, Tongji University. shRNA constructs were generated by inserting target sequences into a pGIPZ backbone digested with XhoI and EcoRI, using ClonExpress® II One Step Cloning Kit. All the target sequences are shown in the table S1. Plasmid transfection was conducted using Lipofectamine 3000 transfection reagent from Invitrogen (Carlsbad, CA, USA) according to the manufacturer’s instructions.

**Antibodies and reagents**

The primary antibodies used in this study are as follows: FUT2 (rabbit, Thermo Fisher Scientific, PA5-53159, 1:200 for IHC or IF), FUT2 (mouse, Santa Cruz Biotechnology, sc-100742, 1:200 for WB), Ki67 (rabbit, Thermo Fisher Scientific, PA5-19462, 1:200 for IHC), CD8a (rat, Thermo Fisher Scientifi, 14-0081-82, 1:200 for IF), β-Actin (mouse, Abmart, 1:5000 for WB), LCN2 (rabbit, Abcam, ab63929, 1:200 for IHC or IF, 1:1000 for WB), NR2F2 (mouse, R&D Systems, PP-H7147-00, 1:1000 for WB), FLAG (rabbit, Abbkine, ABT2011, 1:4000 for WB), HA (rabbit, Cell Signaling Technology, 3724S, 1:1000 for WB), FBXO2 (rabbit, Proteintech, 14590-1-AP, 1:2000 for WB), SKP1 (rabbit, Cell Signaling Technology, 2156, 1:1000 for WB), METTL14 (rabbit, Proteintech, HPA038002, 1:2000 for WB), m6A (rabbit, Synaptic Systems, 202 003, 1:2000 for dot blot), TFEB (rabbit, Proteintech, 13372-1-AP, 1:1000 for WB), TEAD3 (rabbit, Proteintech, 13120-1-AP, 1:1000 for WB), CTNNBIP1 (rabbit, Abcam, ab129011, 1:1000 for WB), ZNF865 (rabbit, Thermo Fisher Scientific, PA5-49280, 1:1000 for WB), METTL3 (rabbit, Proteintech, 15073-1-AP, 1:2000 for WB), METTL16 (rabbit, Proteintech, 19924-1-AP, 1:1000 for WB), WTAP (mouse, Proteintech, 60188-1-Ig, 1:4000 for WB), VIRMA (rabbit, Proteintech, 25712-1-AP, 1:1000 for WB), GST-tag (mouse, ABclonal, AE001, 1:5000 for WB), His-tag (mouse, Proteintech, 66005-1-Ig, 1:5000 for WB), Cleaved caspase-3 (rabbit, Cell Signaling Technology, 9664S, 1:1000 for IHC or WB).

The secondary antibodies goat anti-mouse IgG HRP (G-21040), goat anti-rabbit HRP (G-21234), Donkey anti-rabbit Alexa Fluor™ Plus 488 (A32790) and Donkey anti-rat Alexa Fluor™ Plus 555 (A48270) were purchased from Thermo Fisher Scientific (Shanghai, China).

Reagents: AnnexinV-FITC/PI apoptosis detection kit (556547, BD). TransDectect double-luciferase reporter assay kit (RF201, TransGen Biotech). GDP-Glo^TM^ Glycosyltransferase Assay kit (V6961, Promega). 4’,6-diamidino-2-phenylindole (D9542), GenElute mRNA miniprep kit (DMN10) were purchased from Sigma-Aldrich (St. Louis, MO, USA). TUNEL BrightRed Apoptosis Detection Kit (A113-03), ClonExpress® II One Step Cloning Kit (C112), Trizol reagent (R401-01), HiScript reverse transcriptase (R323-01), FastPure Cell/Tissue Total RNA Isolation Kit V2 (RC112), Taq Pro Universal SYBR qPCR Master Mix (Q712-02), Protein A/G Magnetic Beads (PB101-01), Mouse CD8^+^ T Cell Isolation Kit (CS103-01) were purchased from Vazyme (Nanjing, China). Anti-FLAG M2 magnetic beads (M8823), chromatin IP assay kit (17-295), RIPA lysis buffer, puromycin and hygromycin were purchased from Merck/Millipore (Darmstadt, Germany). Lipocalin-2/NGAL Protein (HY-P70658A), MG-132 (HY-13259), Actinomycin D (HY-17559), Cycloheximide (HY-12320), CIA1 (HY-156190A) were bought from MedChemExpress.

**Lentivirus packaging and infection**

The lentivirus was packaged in HEK293T cells transfected with the target plasmids, psPAX2 (Addgene #12260), and pMD2.G (Addgene #12259) at a ratio of 4:3:1 using polyethylenimine as the transfection reagent. All plasmids used were endotoxin-free. Viral supernatants were collected 48 hours post-transfection, filtered through a 0.22-µm membrane, and used to infect KPC or PANC-1 cells in the presence of polybrene for 12–16 hours. The efficiency of overexpression or knockdown was confirmed by western blot.

**Western blot analysis**

Cells were lysed using RIPA buffer, and the protein concentrations were quantified via BCA assay. Equal amounts of protein were separated by 10% or 15% SDS-PAGE and transferred onto a polyvinylidene difluoride (PVDF) membrane (Millipore, Billerica, MA). The membrane was blocked with 5% non-fat milk at room temperature for 1 hour, followed by incubation with primary antibodies overnight at 4°C. HRP-conjugated secondary antibodies were added and incubated for 1 hour at room temperature. Immunoreactive bands were detected using enhanced chemiluminescence (Thermo Fisher Scientific, Waltham, MA) and visualized with the LAS 4000 Imaging System (Fujifilm, Tokyo, Japan).

**RNA extraction and quantitative RT-PCR (qPCR)**

Total RNA was extracted using TRIzol reagent following the manufacturer’s instructions and reverse-transcribed into cDNA with HiScript Reverse Transcriptase. qPCR was conducted on a real-time system using Taq Pro Universal SYBR qPCR Master Mix, with *ACTB* serving as the internal control. The primer sequences are listed in the Table S2.

**Colony formation assay**

A total of 800 KPC cells per well were plated in six-well plates and incubated at 37°C with 5% CO2. Following treatment with various doses of radiotherapy, the cells were cultured for an additional 7–10 days. The resulting colonies were carefully rinsed with PBS, fixed in 4% paraformaldehyde for 30 min, and stained with crystal violet (Beyotime, Cat. No. C0121) for 40 min. The stained colonies were then imaged and quantified.

**Enzyme activity assay**
The overexpressing FUT2-Flag in KPC or PANC-1 cell were enriched by Anti-Flag M2 magnetic beads and the enzyme activity was measured using a UDP-Glo^TM^ Glycosyltransferase Assay kit (V6961) according to the manufacturer’s protocol. Reagents and a set of standards were prepared for the assays.

**Chromatin immune-precipitation (ChIP) assay**

The ChIP assay was conducted using a chromatin immunoprecipitation assay kit following the manufacturer’s protocol. Briefly, cells were crosslinked with 1% formaldehyde at 37°C, and the reaction was quenched with 125 mM glycine. After washing twice with cold PBS, the cells were processed according to the kit instructions. An anti-NR2F2 antibody was utilized for the ChIP assays, with rabbit IgG serving as a negative control. The enriched DNA of NR2F2 binding to the LCN2 promoter was assessed by qPCR. The primer sequences used for ChIP-qPCR are: Forward 5'-GTGCTGAGCTAGAAGGGTGG, Reverse 5’- CTGTCCCCTCAGGTCCCTAT.

**Dual-luciferase reporter assay**

The promoters of LCN2 were cloned and inserted into the pGL3 basic vector. Cells were co-transfected with the reporter plasmid, the targeting plasmid and the Renilla internal control plasmid. After 48 hours, the cells were lysed, and the supernatant was analyzed using the TransDetect Double-Luciferase Reporter Assay Kit (TransGen Biotech, no. RF201) according to the manufacturer’s instructions.

**Dot blot**

Total RNA was extracted using TRIzol reagent following the manufacturer’s instructions. The RNA pellet was resuspended in RNase-free water, and mRNA was isolated from total RNA using the GenElute mRNA miniprep kit following the manufacturer’s instructions. Isolated mRNA was denatured at 95°C for 5 min, chilled on ice, and dropped onto a Hybond-N+ membrane. After air-drying, RNA was crosslinked to the membrane using UV irradiation (254 nm wavelength, 5 min). The membrane was blocked with 5% non-fat milk in PBST for 1 h, followed by overnight incubation with anti-m6A antibody at 4°C. HRP-conjugated secondary antibodies were added and incubated for 1 hour at room temperature. Chemiluminescent detection was performed using ECL substrate, and the membrane was subsequently stained with 0.02% methylene blue for normalization.

**Table S1.** **The sequences of shRNA**

| Identifier | Sequences (5'-3') |
| --- | --- |
| Human-shMETTL14 | TCTTCTGTACAACCAGTCA |
| Mouse-shMETTL14 | TTCTCATTCGCAGTGATGC |
| Human-shFUT2 | TGATGTTGAGGCTAGCACT |
| Mouse-shFUT2 | GTGTCGCTGTGTAACACCG |
| Human-shFBXO2 | TAACGGTGAGCTCGTAGAG |
| Mouse-shFBXO2  Human-shNR2F2  Mouse-shNR2F2 | CACACTCTCTGGATCACCA  TAACAATTCAAGAACTAAG  TGAACCACAGAATAAACTG |

**Table S2. Quantitative real-time qPCR primers**

| Gene name | Forward (5'-3') | Reverse (5'-3') |
| --- | --- | --- |
| *Mouse-ACTB* | GGCTGTATTCCCCTCCATCG | CCAGTTGGTAACAATGCCATGT |
| *Mouse-LCN2* | GCAGGTGGTACGTTGTGGG | CTCTTGTAGCTCATAGATGGTGC |
| *Mouse-NR2F2* | TCAACTGCCACTCGTACCTG | CCATGATGTTGTTAGGCTGCAT |
| *Mouse-FUT2*  *Mouse-METTL14* | ACCTCCAGCAACGAATAGTGA  TCAAAGGAACCGTGAAGCGA | GCCGATGGAATTGATCGTGAA  CTCCCAAAGAGATGAAGGCGT |
| *Human-ACTB* | CTTCCAGCCTTCCTTCCTGG | TCTTCATTGTGCTGGGTGCC |
| *Human-LCN2* | GAAGTGTGACTACTGGATCAGGA | ACCACTCGGACGAGGTAACT |
| *Human-NR2F2* | CGGGTGGTCGCCTTTATGG | ACAGGCATCTGAGGTGAACAG |
| *Human-FUT2*  *Human-METTL14* | CACCGATGCTGGAAGGGTTT  TGAAGCGTAGCACAGACGG | GAACGACCAGCATGGCTTCT  GGTCCAACTGTGAGCCAGC |

**Table S3. Clinico-characteristics of human pancreatic cancer patients**

| **Human PDAC Patients (N=40)** | | | |
| --- | --- | --- | --- |
| **Gender no. (%)** |  |  |  |
| Male |  | 22 (56.7) |  |
| Female |  | 18 (43.3) |  |
| **Age at enrollments (y)** | | |  |
| Mean (SD) | | 66.5 (4.8) |  |
| Range |  | 45-88 |  |
| **History of Diabetes** |  |  |  |
| Yes |  | 11 (27.5) |  |
| No |  | 29 (72.5) |  |
| **History of Cancer** | |  |  |
| Yes | | 8 (20) |  |
| No | | 32 (80) |  |
| **Operation** | |  |  |
| Pancreaticoduodenectomy | | 31 (77.5) |  |
| Distal pancreatectomy | | 4 (10) |  |
| Biopsy | | 5 (12.5) |  |
| **Early Stage** | | 17 (42.5) |  |
| **Late Stage** | | 23 (57.5) |  |
| **Tumor size, cm^2^** | | 2.7 (2.1-3.6) |  |
| **Extrapancreatic invasion** | |  |  |
| Yes | | 37 (92.5) |  |
| No | | 3 (7.5) |  |
| **Liver metastasis** | |  |  |
| Yes | | 13 (32.5) |  |
| No | | 27 (67.5) |  |
| **Local recurrence** | |  |  |
| Yes | | 27 (67.5) |  |
| No | | 13 (32.5) |  |
| **T stage no. (%)** | |  |  |
| T1 |  | 1 (2.5) |  |
| T2 |  | 21 (57.5) |  |
| T3 |  | 13 (27.5) |  |
| T4 |  | 5 (12.5) |  |
| **N stage, no. (%)** | |  |  |
| N0 | | 13 (32.5) |  |
| N1 |  | 15 (37.5) |  |
| N2 |  | 12 (30) |  |
| **Differentiation** |  |  |  |
| Well/Moderately differentiated |  | 34 (85) |  |
| Poorly differentiated |  | 6 (15) |  |
