# Supplementary material for: FUT2 enhances anti-tumor immunity in pancreatic cancer radiotherapy by driving FBXO2-mediated degradation of NR2F2
Source: Cell Death Dis. 2025 Dec 23;17(1):126. doi: 10.1038/s41419-025-08378-2 (PMC12848027; doi:10.1038/s41419-025-08378-2)
Supplement: Supplementary file 2 — Western Blotting Original Image [file 41419_2025_8378_MOESM2_ESM.pdf]

Fig. S1

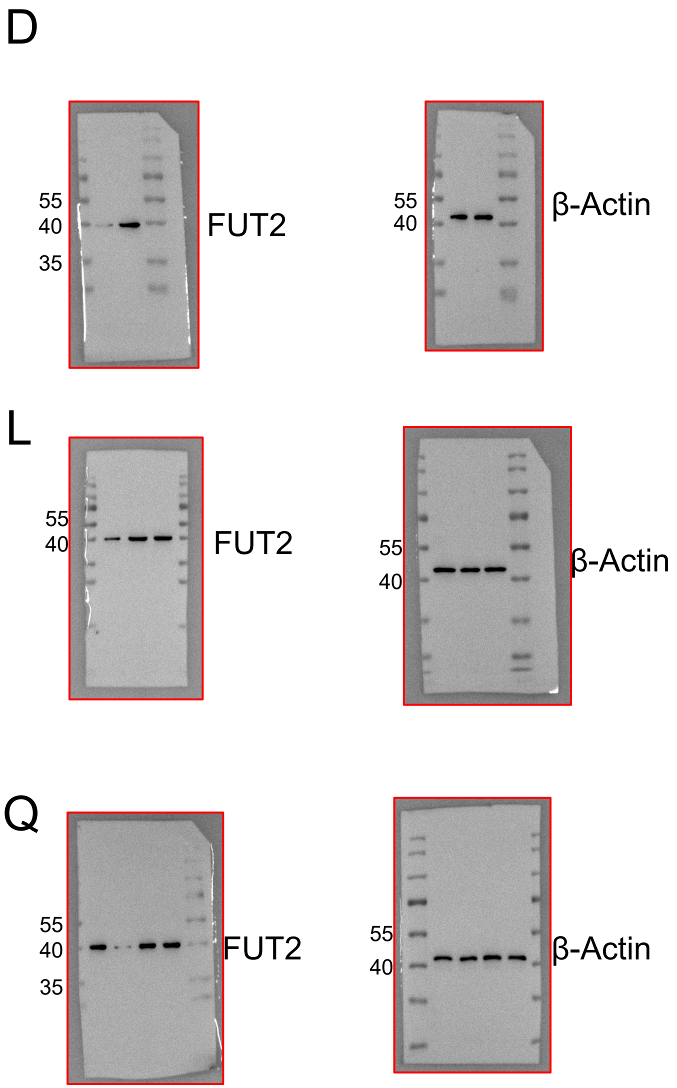
D
55
40
35
FUT2
55
40
β-Actin
L
55
40
FUT2
55
40
β-Actin
Q
55
40
35
FUT2
55
40
β-Actin

Fig. 2

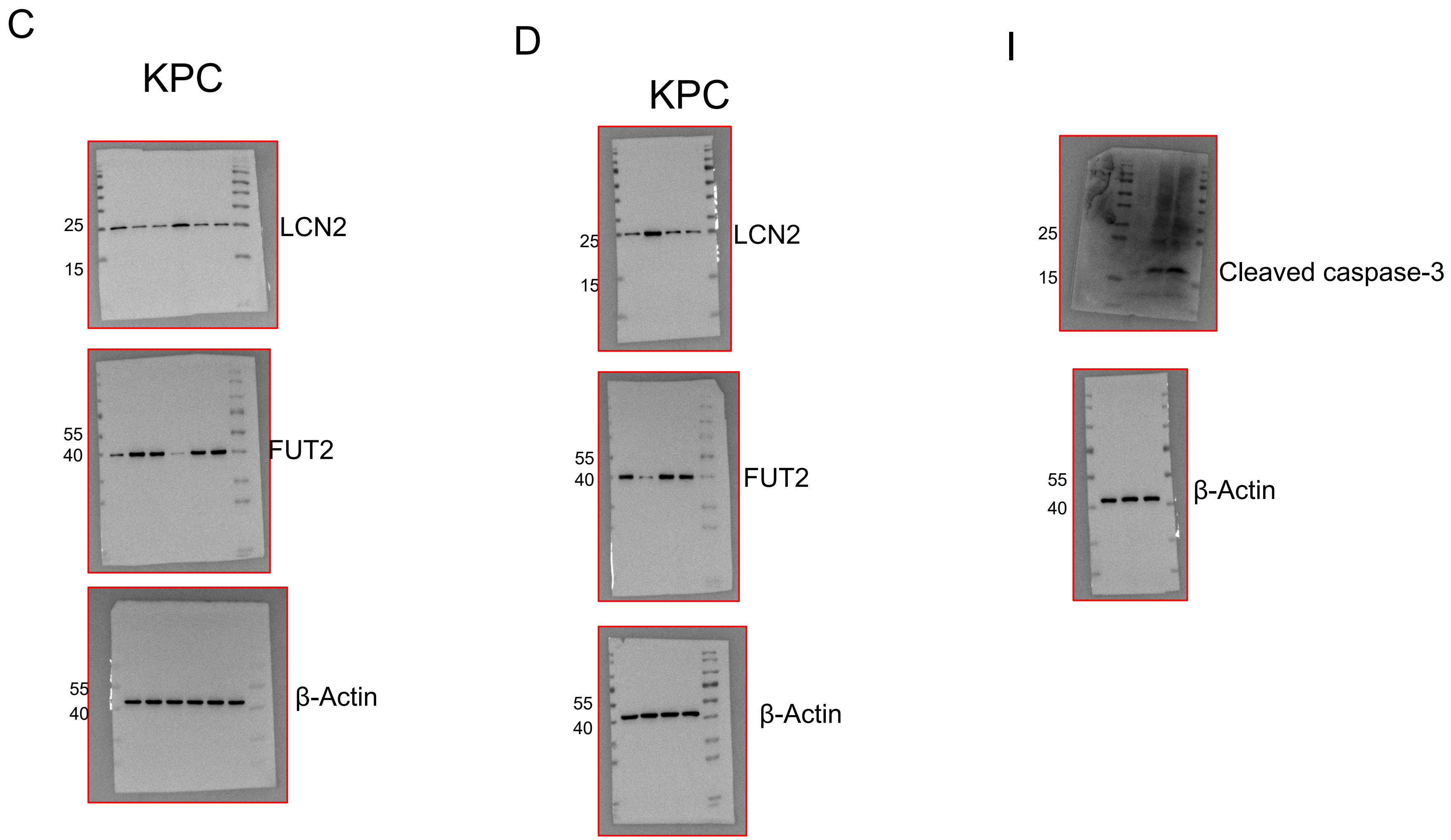
C
KPC
25
15
LCN2
55
40
FUT2
55
40
β-Actin
D
KPC
25
15
LCN2
55
40
FUT2
55
40
β-Actin
I
25
15
Cleaved caspase-3
55
40
β-Actin

Fig. S2

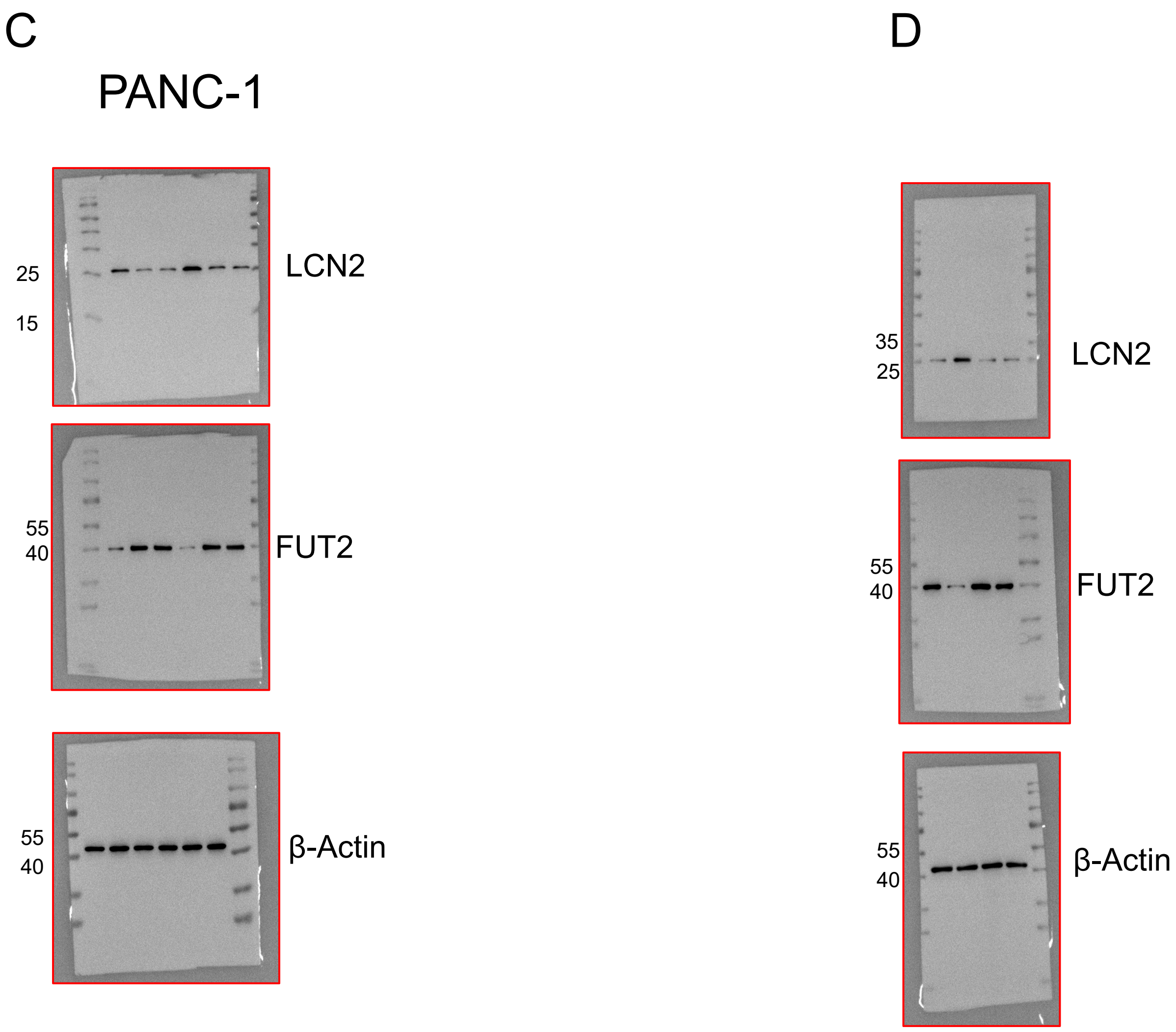
C
PANC-1
25
15
LCN2
55
40
FUT2
55
40
β-Actin
D
35
25
LCN2
55
40
FUT2
55
40
β-Actin

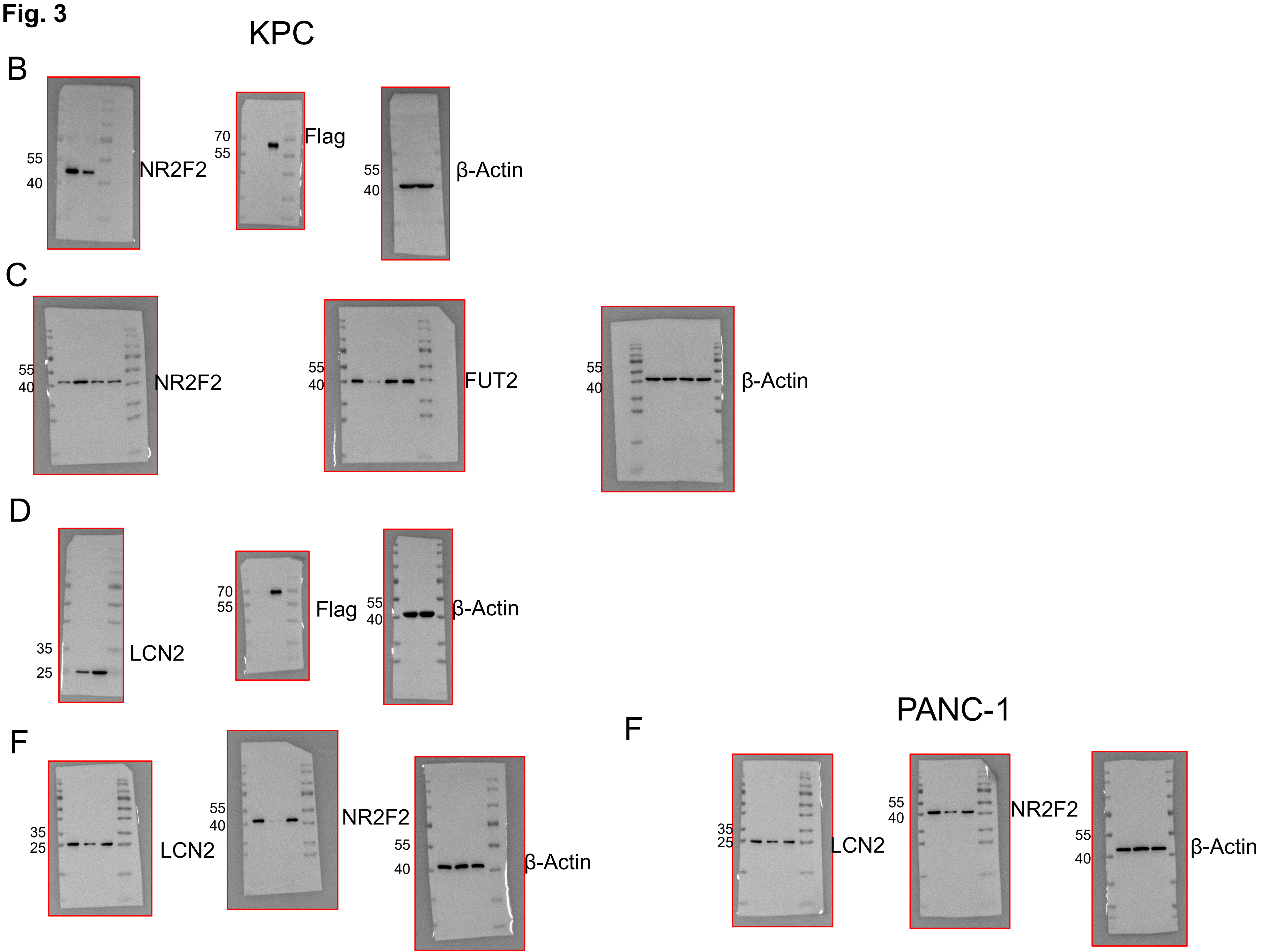
Fig. 3
KPC
B
55
40
NR2F2
70
55
Flag
55
40
β-Actin
C
55
40
NR2F2
55
40
FUT2
55
40
β-Actin
D
35
25
LCN2
70
55
Flag
55
40
β-Actin
F
35
25
LCN2
55
40
NR2F2
55
40
β-Actin
PANC-1
F
35
25
LCN2
55
40
NR2F2
55
40
β-Actin

Fig. 3

H

KPC

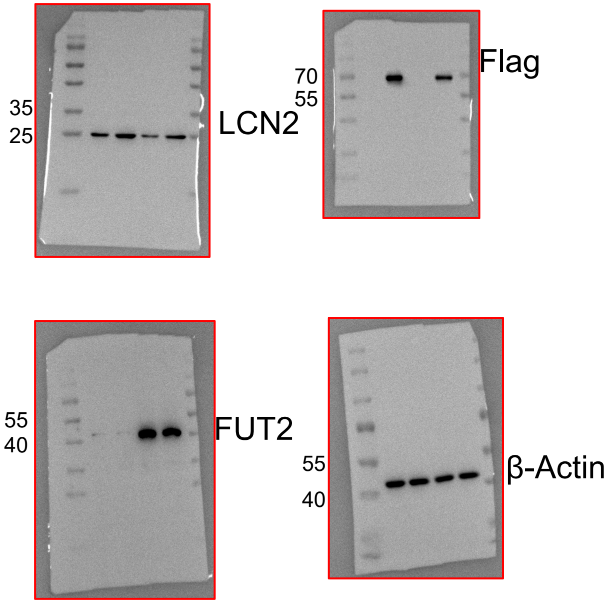

H

PANC-1

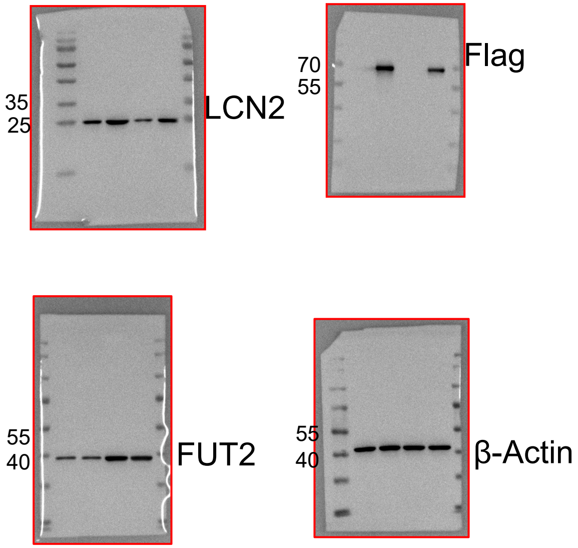

Fig. 3

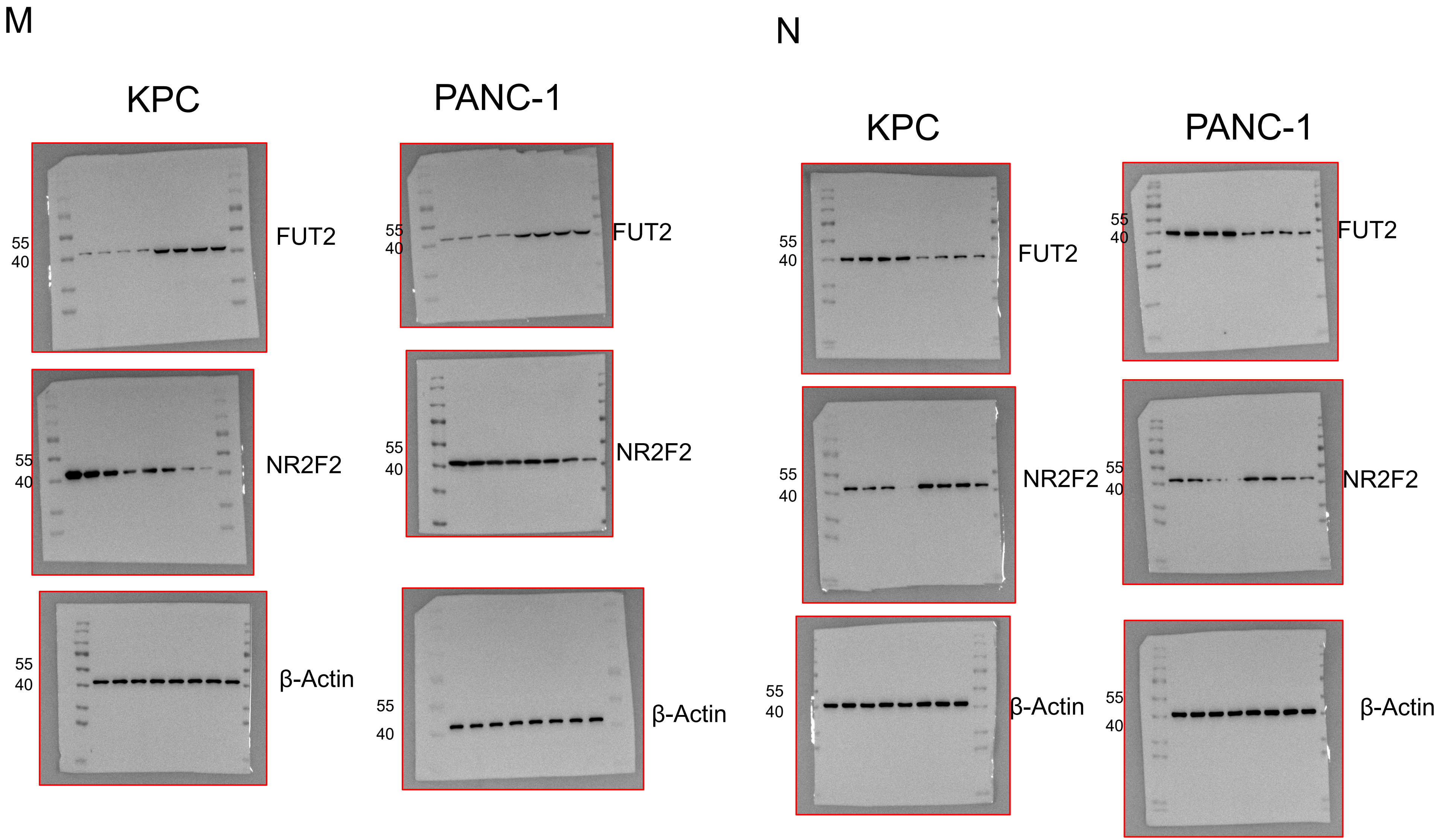
M
KPC
PANC-1
FUT2
NR2F2
β-Actin
55
40
N
KPC
PANC-1
FUT2
NR2F2
β-Actin
55
40

Fig. S3

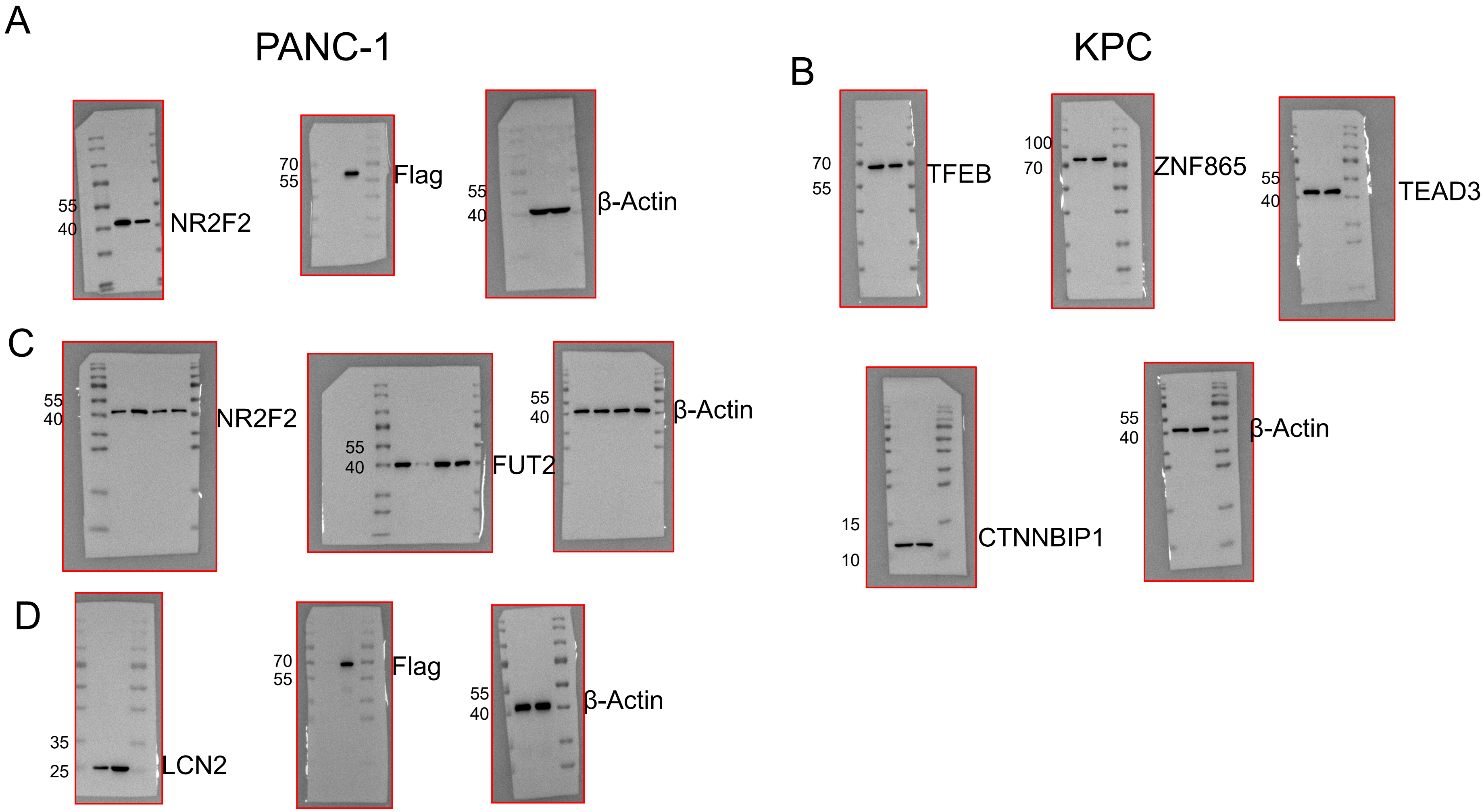
A
PANC-1
55
40
NR2F2
70
55
Flag
55
40
β-Actin
B
KPC
70
55
TFEB
100
70
ZNF865
55
40
TEAD3
C
55
40
NR2F2
55
40
FUT2
55
40
β-Actin
15
10
CTNNBIP1
55
40
β-Actin
D
35
25
LCN2
70
55
Flag
55
40
β-Actin

Fig. 4

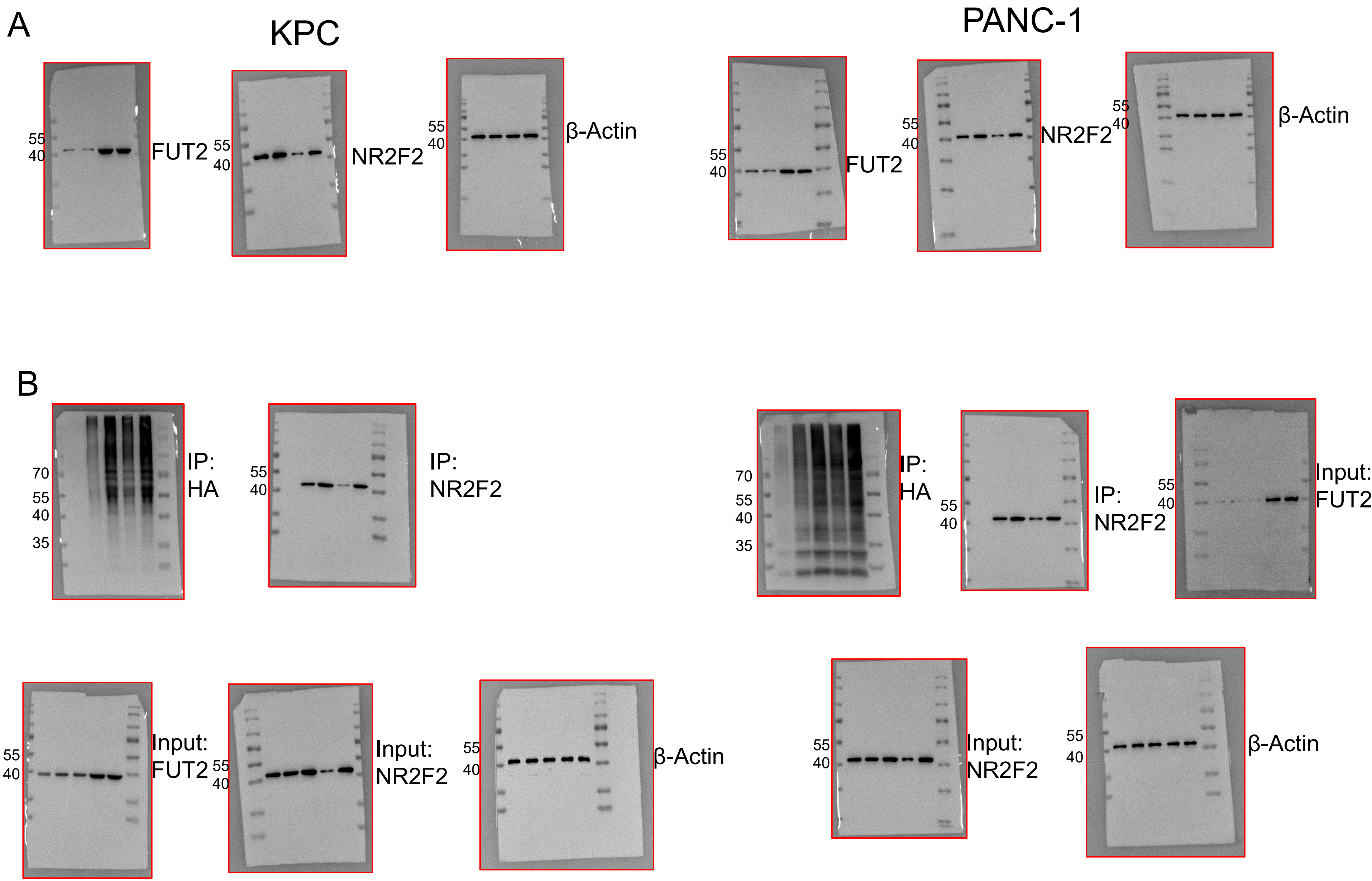
A
KPC
PANC-1
55
40
FUT2
55
40
NR2F2
55
40
β-Actin
55
40
FUT2
55
40
NR2F2
55
40
β-Actin
B
70
55
40
35
IP:
HA
55
40
IP:
NR2F2
70
55
40
35
IP:
HA
55
40
IP:
NR2F2
55
40
Input:
FUT2
55
40
Input:
FUT2
55
40
Input:
NR2F2
55
40
β-Actin
55
40
Input:
NR2F2
55
40
β-Actin

Fig. 4

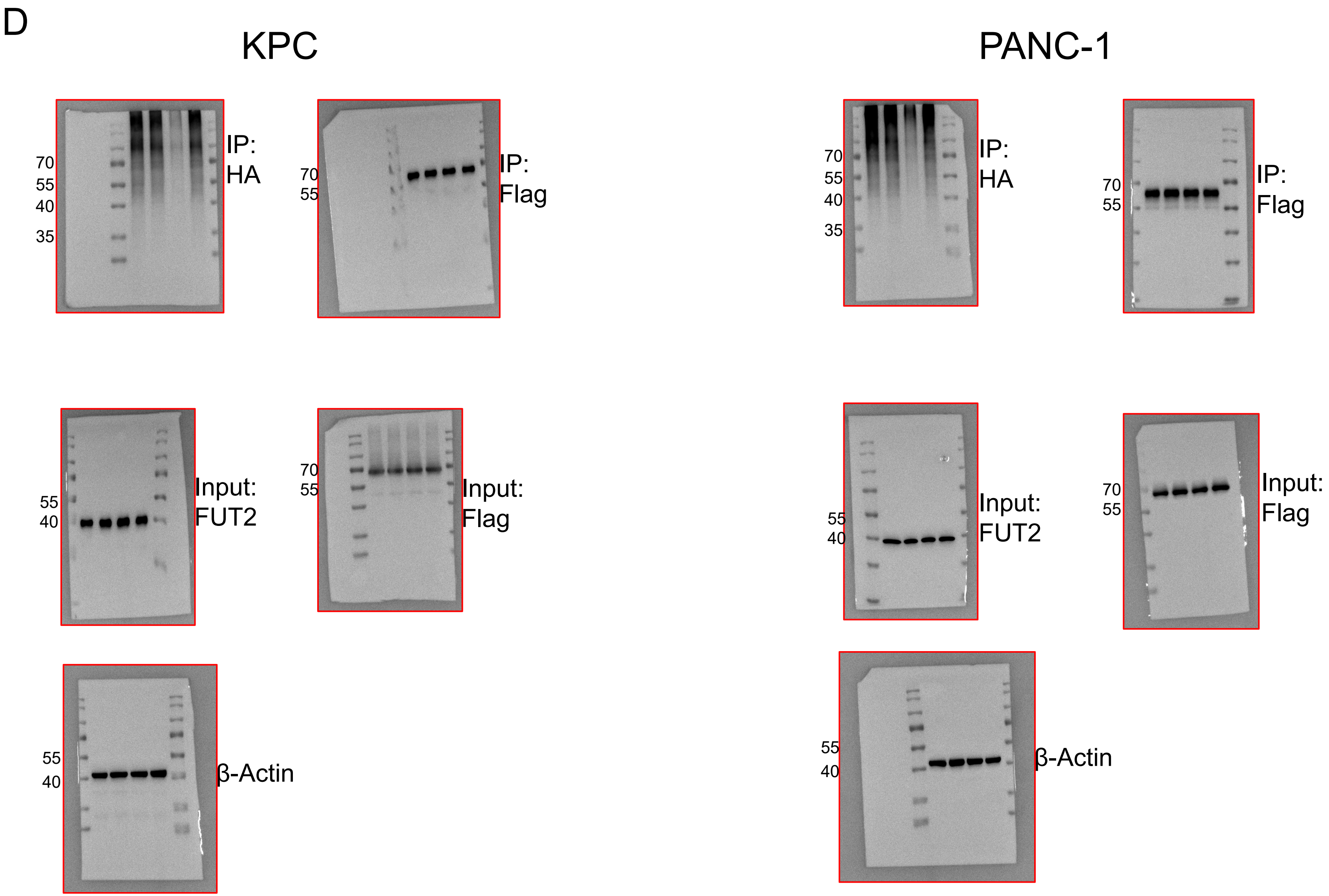
D
KPC
PANC-1
70
55
40
35
IP:
HA
70
55
IP:
Flag
55
40
Input:
FUT2
70
55
Input:
Flag
55
40
β-Actin
70
55
40
35
IP:
HA
70
55
IP:
Flag
55
40
Input:
FUT2
70
55
Input:
Flag
55
40
β-Actin

Fig. S4

A

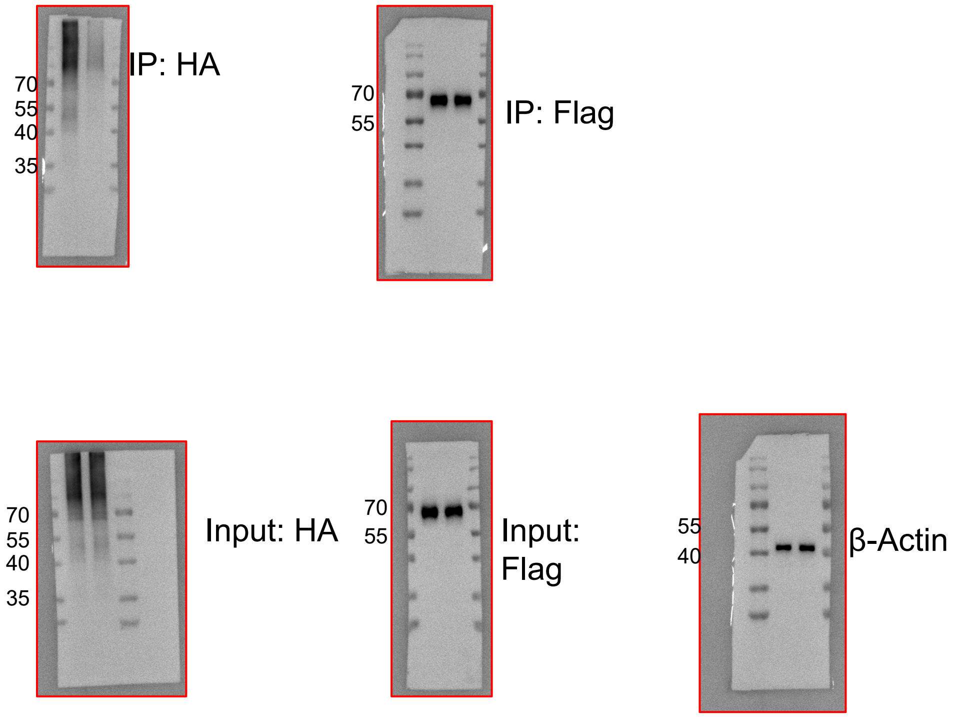

IP: HA
70
55
40
35
IP: Flag
70
55
Input: HA
70
55
40
35
Input: Flag
70
55
β-Actin
55
40

Fig. 5

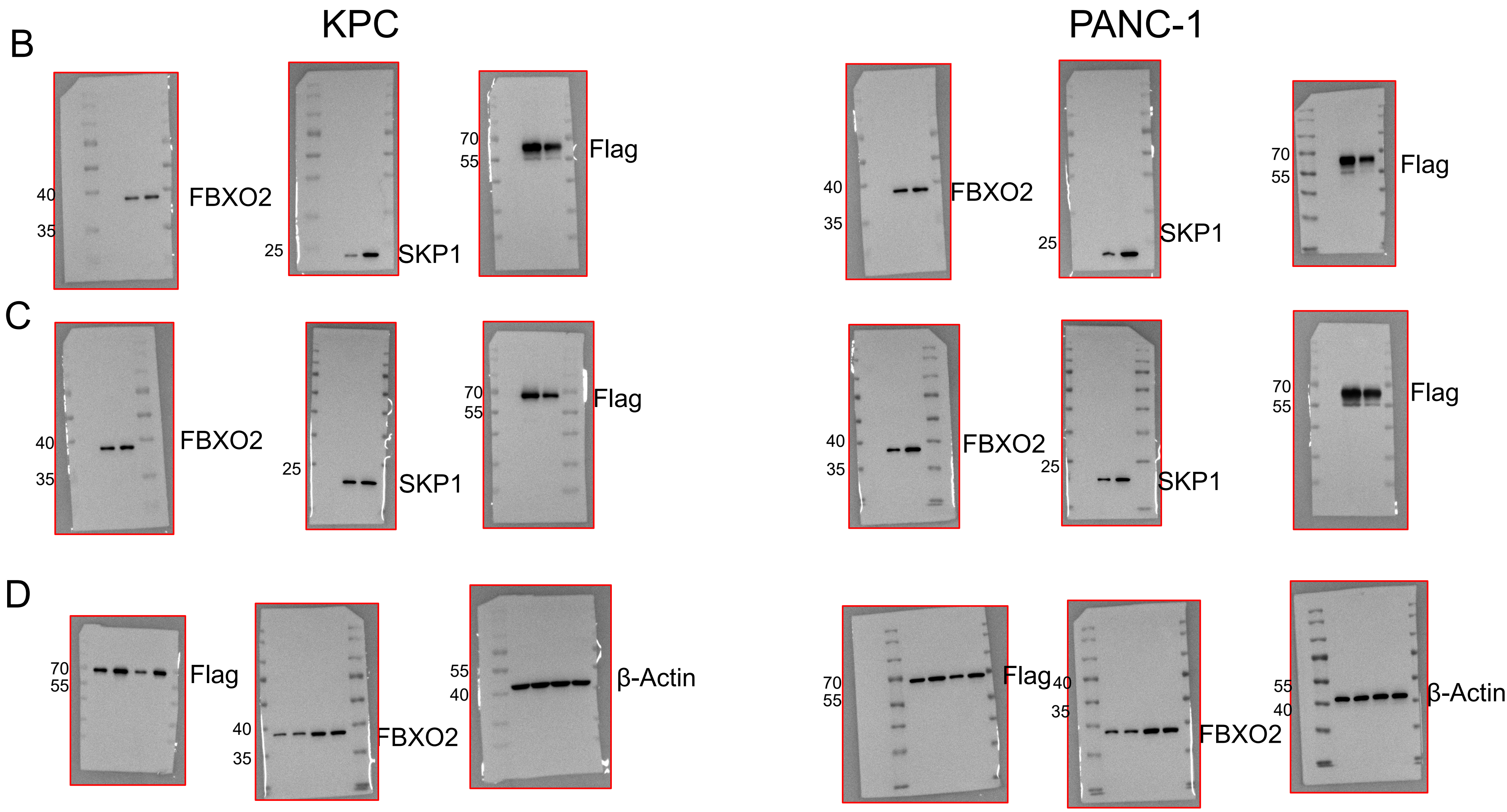

Fig. 5

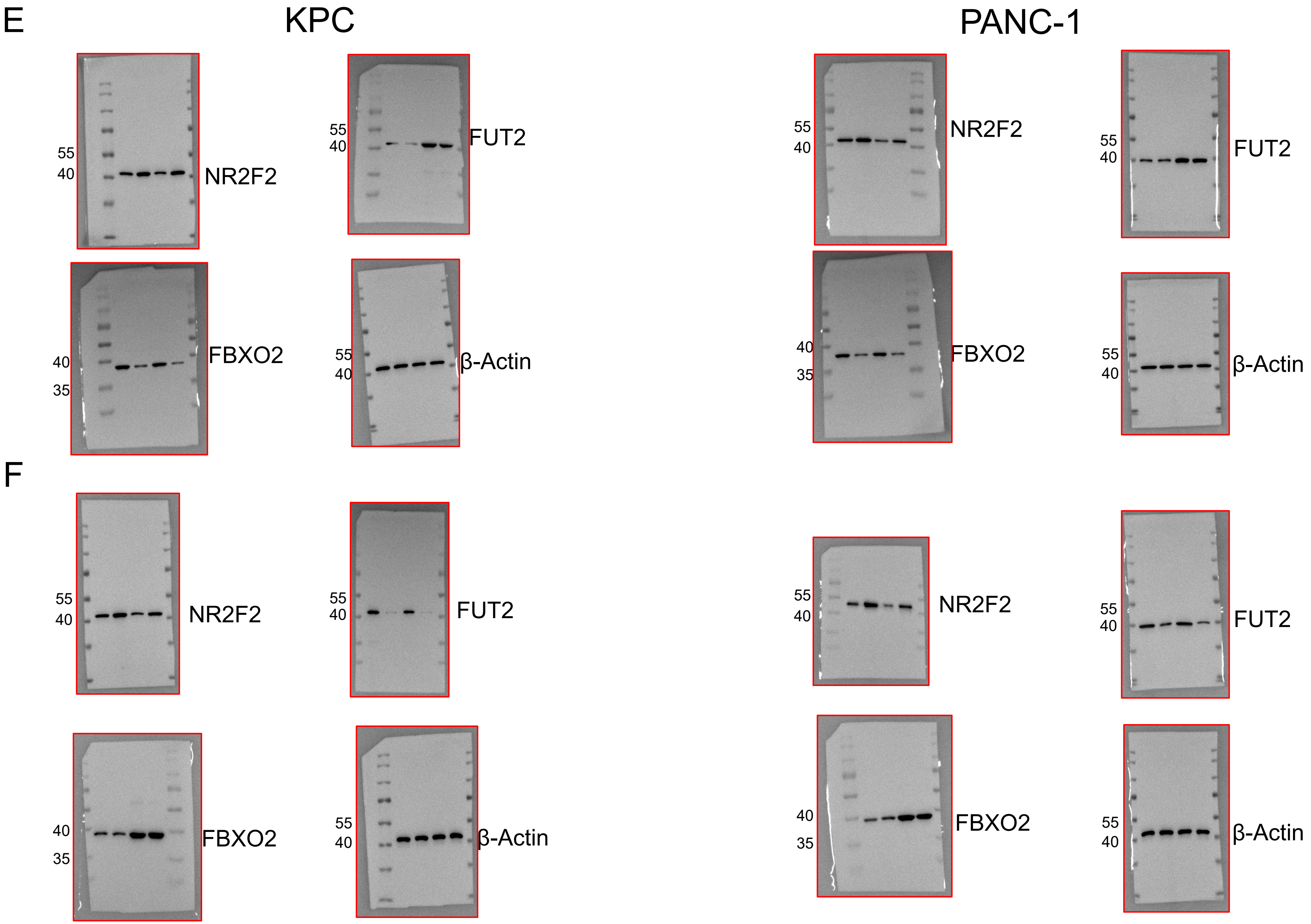
E
KPC
PANC-1
55
40
NR2F2
55
40
FUT2
40
35
FBXO2
55
40
β-Actin
F
55
40
NR2F2
55
40
FUT2
40
35
FBXO2
55
40
β-Actin

Fig. 5

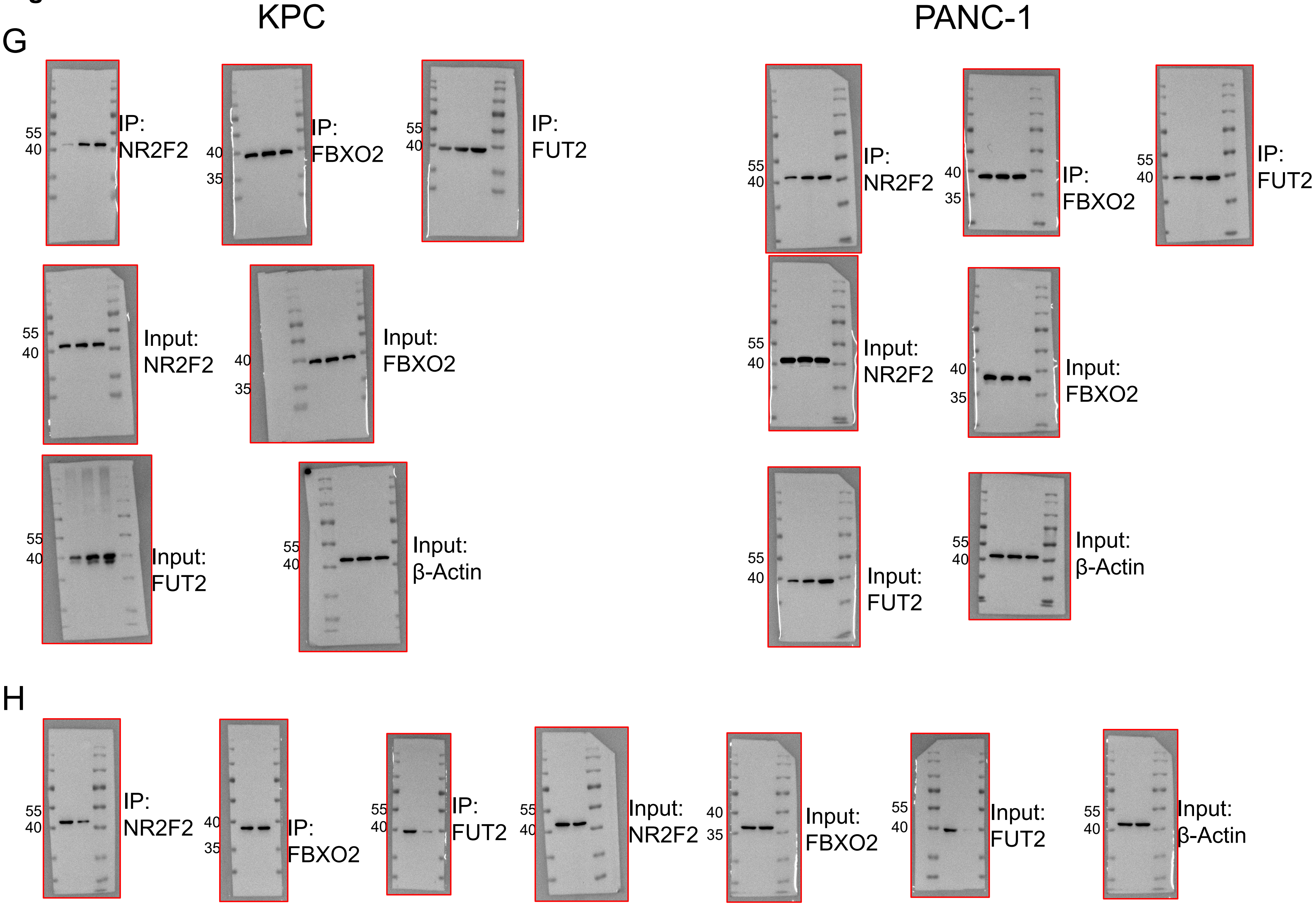
G
KPC
PANC-1
IP: NR2F2
IP: FBXO2
IP: FUT2
Input: NR2F2
Input: FBXO2
Input: FUT2
Input: β-Actin
H
IP: NR2F2
IP: FBXO2
IP: FUT2
Input: NR2F2
Input: FBXO2
Input: FUT2
Input: β-Actin
55
40
35

Fig. 5

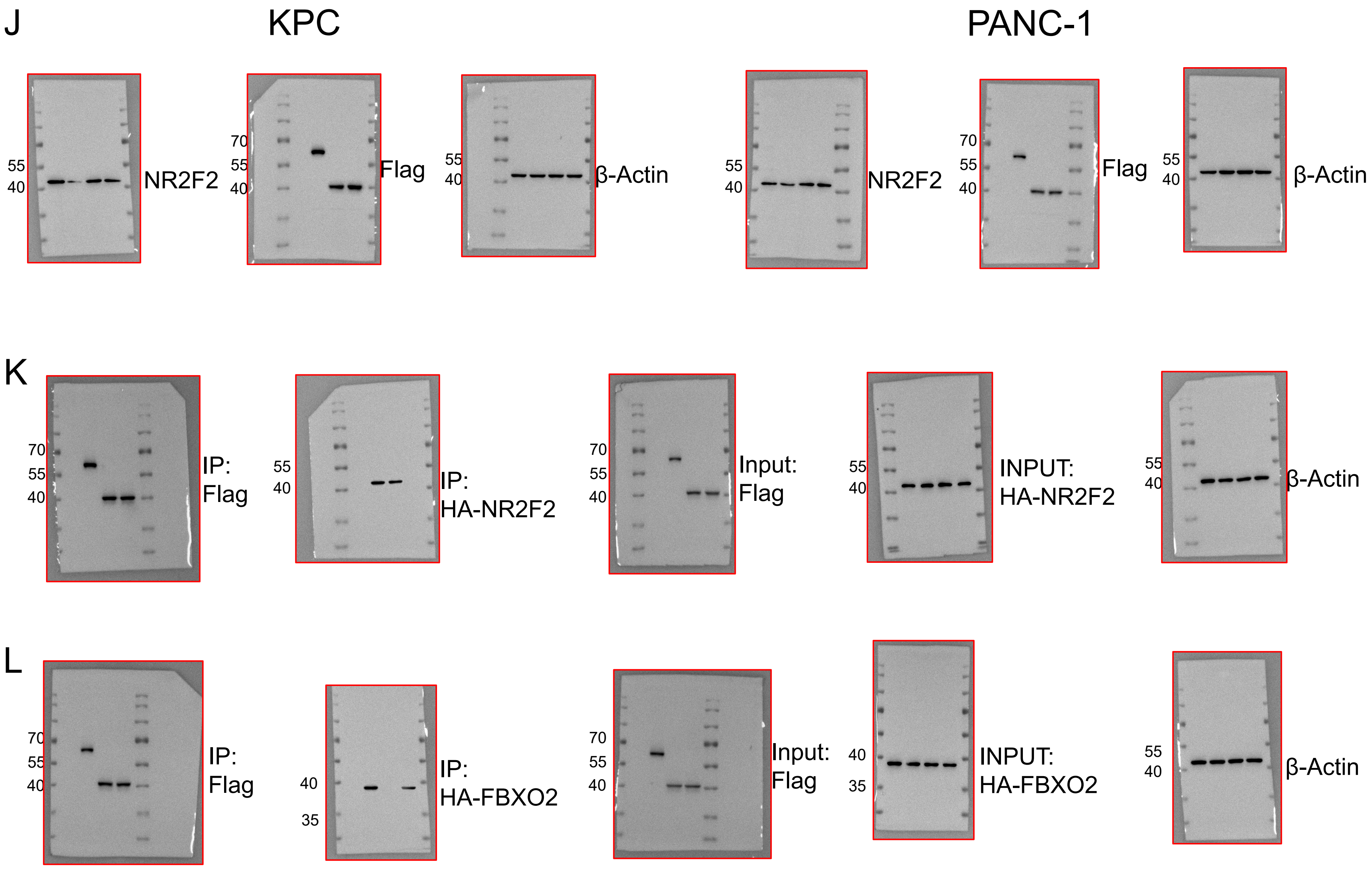
J
KPC
PANC-1
55
40
NR2F2
70
55
40
Flag
55
40
β-Actin
55
40
NR2F2
70
55
40
Flag
55
40
β-Actin
K
70
55
40
IP:
Flag
55
40
IP:
HA-NR2F2
70
55
40
Input:
Flag
55
40
INPUT:
HA-NR2F2
55
40
β-Actin
L
70
55
40
IP:
Flag
40
35
IP:
HA-FBXO2
70
55
40
Input:
Flag
40
35
INPUT:
HA-FBXO2
55
40
β-Actin

Fig. S5

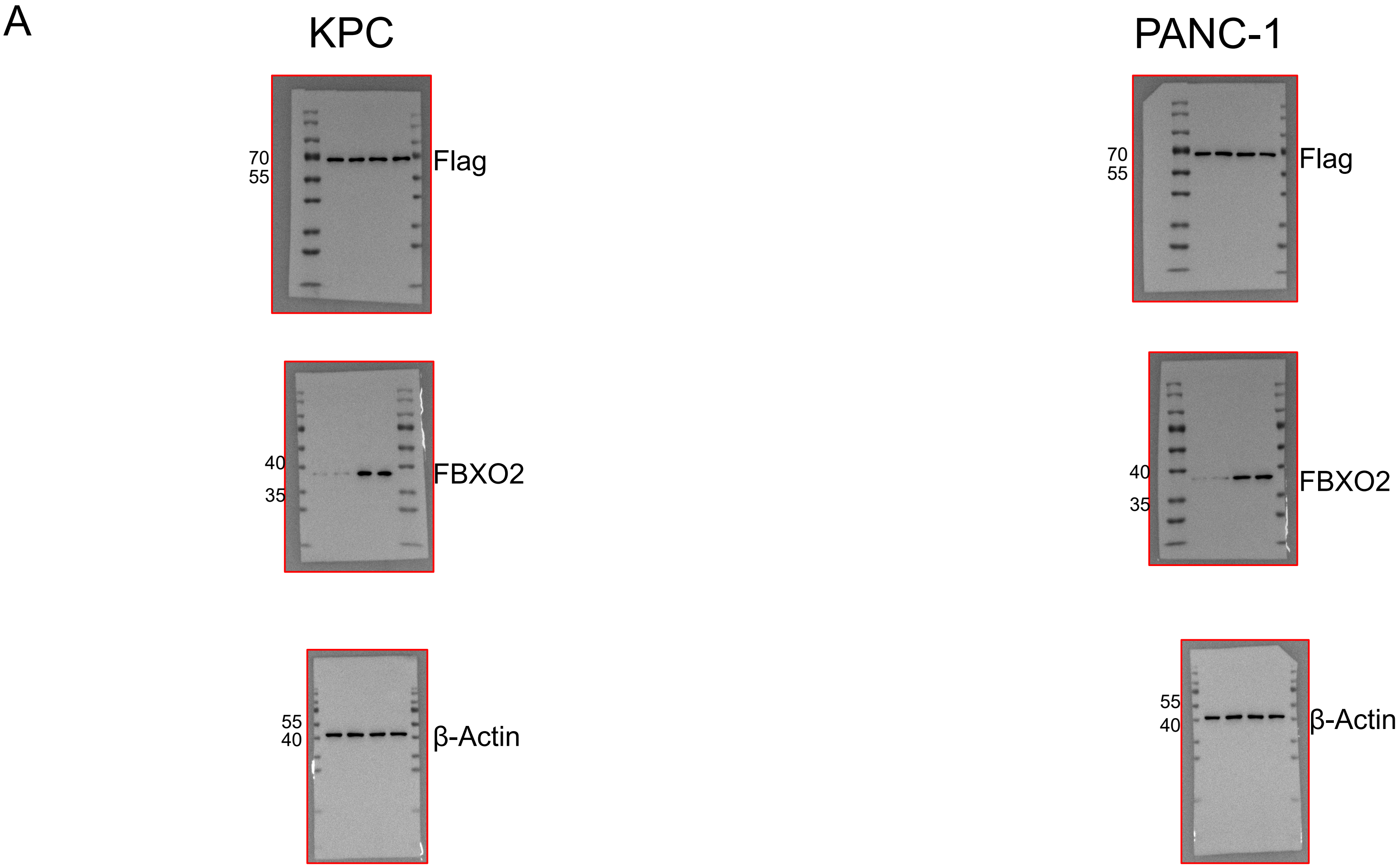
A
KPC
PANC-1
70
55
Flag
40
35
FBXO2
55
40
β-Actin
70
55
Flag
40
35
FBXO2
55
40
β-Actin

Fig. S5

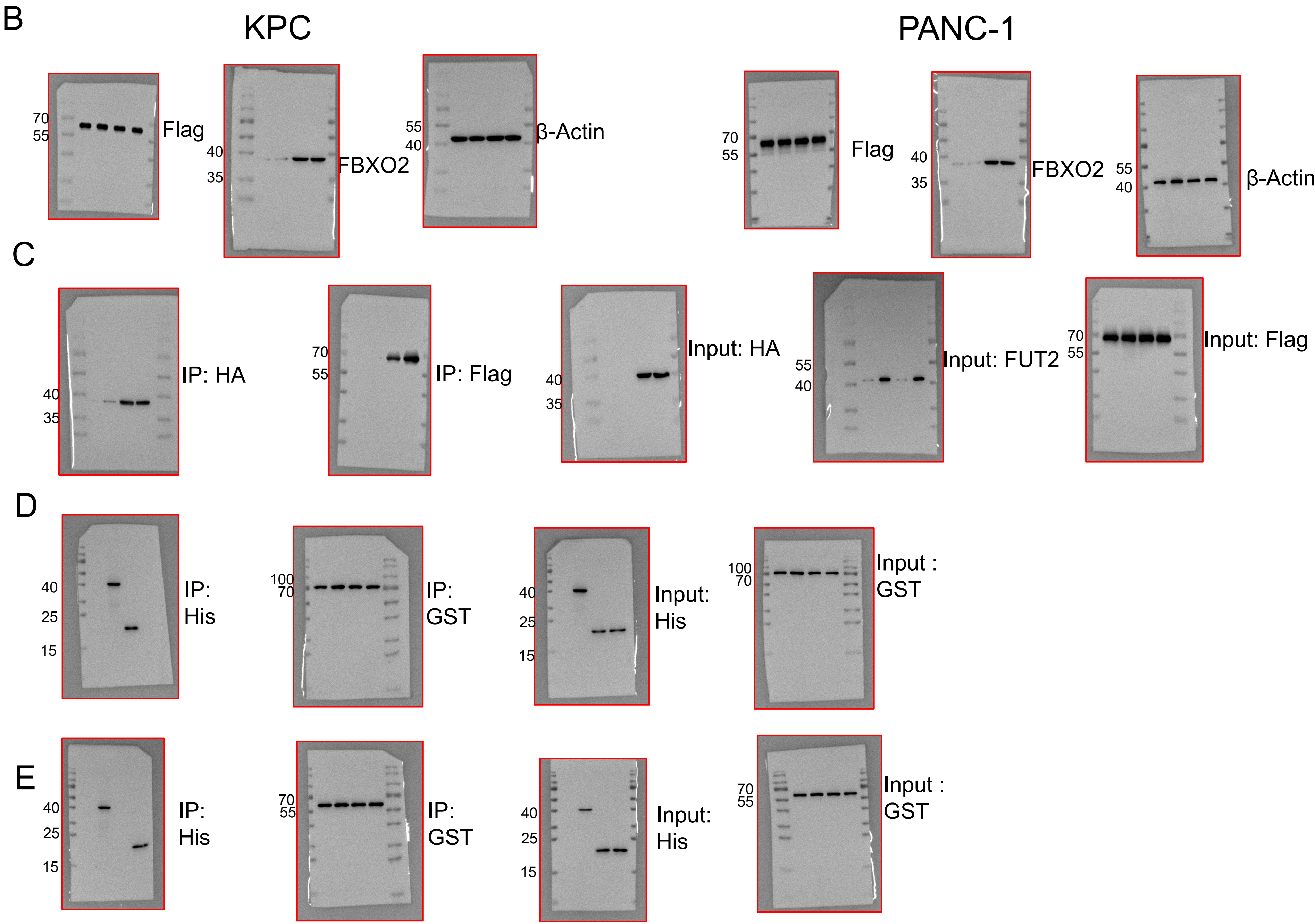
B
KPC
PANC-1
70
55
Flag
40
35
FBXO2
55
40
β-Actin
C
IP: HA
IP: Flag
Input: HA
Input: FUT2
Input: Flag
D
IP:
His
IP:
GST
Input:
His
Input :
GST
100
70
25
15
E

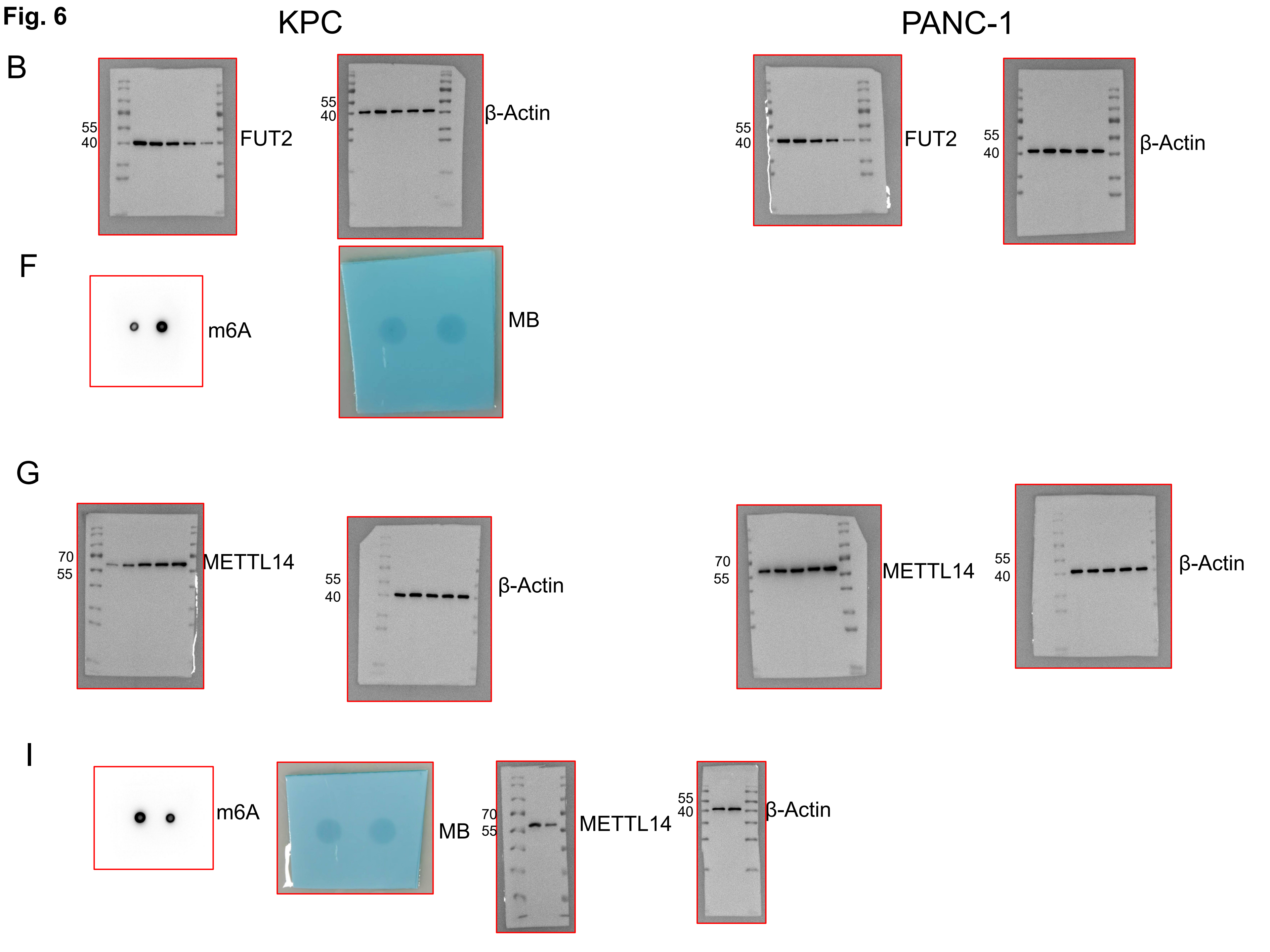
Fig. 6
KPC
PANC-1
B
55
40
FUT2
55
40
β-Actin
55
40
FUT2
55
40
β-Actin
F
m6A
MB
G
70
55
METTL14
55
40
β-Actin
70
55
METTL14
55
40
β-Actin
I
m6A
MB
70
55
METTL14
55
40
β-Actin

Fig. S6

B

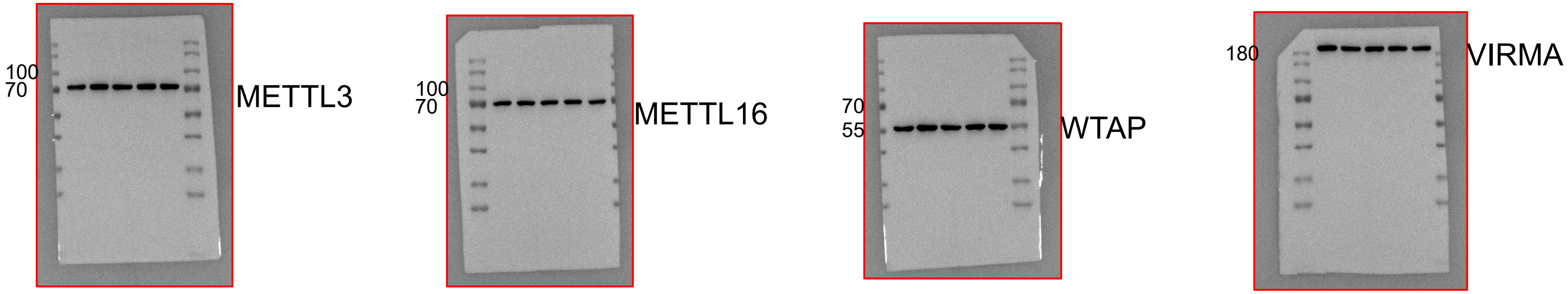
100
70
METTL3
100
70
METTL16
70
55
WTAP
180
VIRMA

E

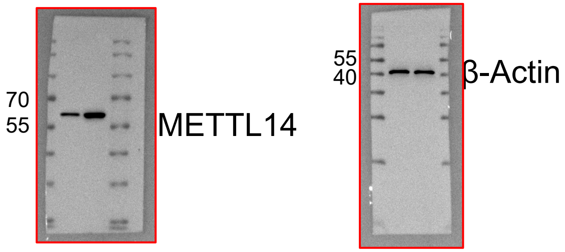
70
55
METTL14
55
40
β-Actin
